# Alkylamide Profiling of Pericarps Coupled with Chemometric Analysis to Distinguish Prickly Ash Pericarps

**DOI:** 10.3390/foods10040866

**Published:** 2021-04-15

**Authors:** Yao Ma, Lu Tian, Xiaona Wang, Chen Huang, Mingjing Tian, Anzhi Wei

**Affiliations:** 1 College of Forestry, Northwest A&F University, Yangling 712100, China; mayao277000@nwafu.edu.cn (Y.M.); t1anlu@nwafu.edu.cn (L.T.); 17709590958@nwafu.edu.cn (X.W.); hc19990513@nwafu.edu.cn (C.H.); 15531971892@nwafu.edu.cn (M.T.); 2 Research Centre for Engineering and Technology of Zanthoxylum, State Forestry Administration, Yangling 712100, China

**Keywords:** alkylamide profiling, prickly ash pericarps, influence factors, chemometrics

## Abstract

Because of extensive cultivation areas, various cultivars, nonstandard naming notations, and morphology similarity among relative cultivars, adulteration and associated business fraud may happen in the marketplaces of prickly ash pericarps due to higher financial gain and high-frequency trading. This study presents variations in the chemical components and contents of different prickly ash species from different plantations. Alkylamide profiling of pericarps derived from *Zanthoxylum armatum*, *Z. bungeanum,* and some relative *Zanthoxylum* species from 72 plantations across China were tested using ultra-performance liquid chromatography. Then, several chemometrics were applied to classify the prickly ash pericarps to reveal potential indicators that distinguish prickly ash pericarps and to identify the key factors that affect pericarp alkylamide profiling. The dominating alkylamides in the prickly ash pericarps were *Z. piperitum* (ZP)-amide C (0–20.64 mg/g) and ZP-amide D (0–30.43 mg/g). Alkylamide profiling of prickly ash pericarps varied significantly across species and geographical variations. ZP-amide D in prickly ash pericarps was identified as a potential indicator to distinguish prickly ash species. Longitude and aluminum content in soils were identified as key factors that affected alkylamide profiling of prickly ash pericarps. This study provides a useful tool to classify prickly ash species based on pericarp alkylamide profiling and to determine the key influence factors on pericarp alkylamide variations.

## 1. Introduction

In China, prickly ash (*Zanthoxylum*) is an important medicinal and edible homologous plant because of its aroma, taste, and health benefits [1,2,3,4]. The trees are distributed across a variety of regions due to their high adaptation to diverse environmental conditions [5,6,7]. The resources of prickly ash are very rich in China because of regional introduction, breeding and individual variation, and varying environmental conditions [8,9,10]. The overlapping distribution of some prickly ash species, inconsistent nomenclature of species cultivated in different areas, and similarity in morphology of some pericarps cause difficulty in distinguishing pericarp species [8,9,10]. Furthermore, it is more difficult to distinguish pericarps when they are sold as powders. The prickly ash species in China are separated into four categories based on their genetic relationships: prickly ash in first group ZA (*Z. armatum* DC); prickly ash in second group ZB1 (*Z. bungeanum* 1), and consists of samples from Hancheng; prickly ash in third group ZB2 (*Z. bungeanum* 2), but the samples come from Fengxian; and prickly ash in group other with red pericarps, easy to confuse with ZB, contains the remaining samples, which are a mixture of some species, excluding the species of ZB [7,11]. ZA and ZB are two commonly cultivated species, and the pericarps are irreplaceable ingredients in Chinese cuisine due to their special flavors.

Adulteration and business fraud may happen in trading these materials to gain greater financial benefit. Since quality pericarps are expensive and it is difficult to establish pericarp authenticity, unscrupulous dealers may mislabel the geographical location of pericarps, intentionally adulterate the pericarps, or conduct other fraudulent behaviors [11]. Molecular methods are often used to distinguish the species and identify adulterants; these methods are accurate and effective, and they do not depend on collection time, environmental factors, or storage and processing methods [12]. However, extracting DNA or RNA is difficult due to the strict requirements for sampling methods and sample storing. Moreover, the quality of materials derived from the same species vary due to geographical variations [13]. Physical and chemical analyses, using instruments and apparatuses coupled with chemometrics, are available to detect adulterations in food commodities [13,14]. Chromatography methods include liquid chromatography, high/ultra-performance liquid chromatography, liquid chromatography–mass spectrometry, and gas chromatography–mass spectrometry; spectroscopic methods include nuclear magnetic resonance spectroscopy; electrophoretic methods include capillary electrophoresis; and electronic methods include electronic nose and electronic tongue. These methods have been applied to differentiate samples based on their chemical profile variations [15,16,17,18]. Some remarkable differences in chemical compositions (aroma constituents, alkylamides, and volatile oil content) occur in different prickly ash pericarps due to variations in location, climate, and soil conditions [5,9,10,13]. The presence of alkylamides in pericarps contributes to the perception of numb taste when the pericarps are used as spices [19]. Alkylamides are long-chain unsaturated fatty structures, and hydroxyl-α-sanshool (Compound H), hydroxyl-β-sanshool (Compound I), and hydroxyl-γ-sanshool are dominant in prickly ash pericarps [10,13,20,21]. These alkylamides exhibit some bioactivities, such as attenuating learning and memory impairments [22] and protecting corticosterone-treated PC12 cells [23], and have anti-inflammatory properties [24]. The alkylamide profiling variation from different pericarps is not explicit. Thus, an effective approach to determine the authenticity of pericarps in the marketplaces is urgent, and the proposed method is based on alkylamide profiling.

To determine the differences in alkylamide content between pericarps, prickly ash pericarps collected from 72 plantations across China were tested using ultra-performance liquid chromatography (UPLC). Then, several chemometric methods were carried out to better understand the differences among pericarps based on alkylamide profiling. Moreover, the relationship between alkylamide profiling and environmental factors (location, climate, and soil conditions) was analyzed using redundancy analysis (RDA). The proposed approach is a useful tool to determine the alkylamide composition of different prickly ash species and determine the key environmental factors that cause alkylamide variations in pericarps. Hence, this tool will help to improve the quality of pericarps by introducing a better prickly ash cultivar and changing the cultivated environments.

## 2. Materials and Methods

### 2.1. Sample Collection and Preparation

**Figure 1:**
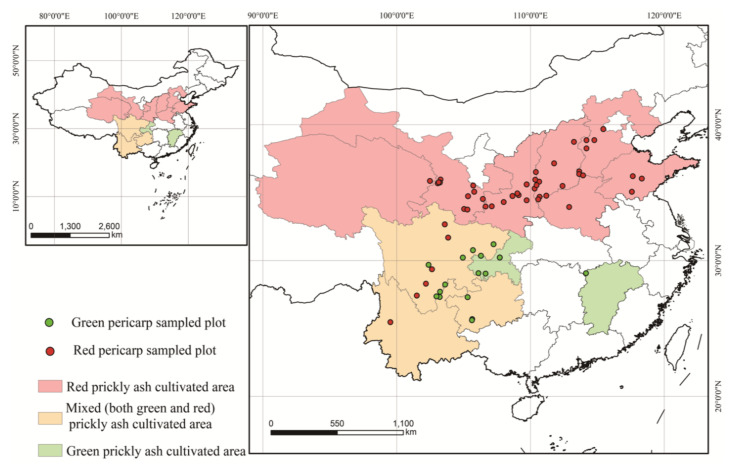
Sample origins for the 72 prickly ash pericarps.

Prickly ash samples were collected from 72 plantations across 12 provinces (Shandong, Hebei, Shanxi, Shaanxi, Henan, Gansu, Qinghai, Sichuan, Chongqing, Guizhou, Jiangxi, and Yunnan). The red pericarp samples (Z1–Z55) were from 55 plantations, and the green pericarp samples (Z56–Z72) were from 17 plantations (Figure 1). About 0.5 kg of mixed topsoil samples (0–5 cm) were collected from five sites on each plantation, and 5 kg of mixed fruit samples were collected randomly from five trees on each plantation. A minimum of three biological repetitions were created for each plantation for testing the fruit and topsoil samples. Information on the samples was recorded (Table S1), and collected samples in valve bags from each plantation were transported to the laboratory. The pericarps were separated from dried fruit, and then dried soil and pericarp samples were ground to a homogenized powder.

### 2.2. Determination of Environmental Factors

The location data on longitude (Long), latitude (Lat), and altitude (Alt) of each plantation were obtained from a GPS real-time altitude app (Fuzhou Lexun Network Technology Co., LTD, Fuzhou, China); the climate data on mean atmospheric pressure (AtP), mean temperature (MT), mean relative humidity (MRH), and mean annual precipitation (MAP) were collected from http://data.cma.cn/ (24 December 2018). Soil organic matter (OM) was detected using an external heating method with potassium dichromate-concentrated sulfuric acid to determine the soil conditions. The power of hydrogen in the soil (pH) was detected using a PB-10 pH meter (Sartorius AG, Goettingen, Germany). The total nitrogen content in the soil (N_t_) was determined using an AutoAnalyzer 3 (Seal Analytical GmbH, Norderstedt, Germany); the available nitrogen content in the soil (N_a_) was detected using alkaline hydrolysis diffusion. The total phosphorus content in the soil (P_t_) and available phosphorus content in the soil (P_a_) were detected using the molybdenum–antimony colorimetric method; the total potassium content in the soil (K_t_) and available potassium content in the soil (K_a_) were detected using flame photometry (Shanghai Precision Science Instrument Co., Ltd. Shanghai, China). The contents of aluminum (Al), cadmium (Cd), lead (Pb), manganese (Mn), and nickel (Ni) in the soil samples were detected using inductively coupled plasma-optical emission spectrometry (PerkinElmer Co., Waltham, MA, USA), and the arsenic (As) content in the soil was detected using an AFS-2100 atomic fluorescence spectrophotometer (Beijing Haiguang Instrument Co. Ltd., Beijing, China) after the digesting of mixed acids. The detailed data on location, climate, and soil are reported in our other studies [25,26] and also shown in Tables S1–S3.

### 2.3. Qualitative and Quantitive Determinations of Prickly Ash Pericarp Alkylamides

Thirty grams of dry pericarp powder samples were extracted using distilled water (300 mL) in a glass instrument and continuously heated to ebullition for 4 h to simulate hot pot progress. After cooling, the solutions were centrifuged to collect liquid supernatants using a TGL-18M high-speed freezing centrifuge (Shanghai Luxiangyi Centrifuge Instrument Co. LTD, Shanghai, China). The liquid supernatants were then diluted to an appropriate concentration to determine the alkylamides in prickly ash pericarps.

**Figure 2:**
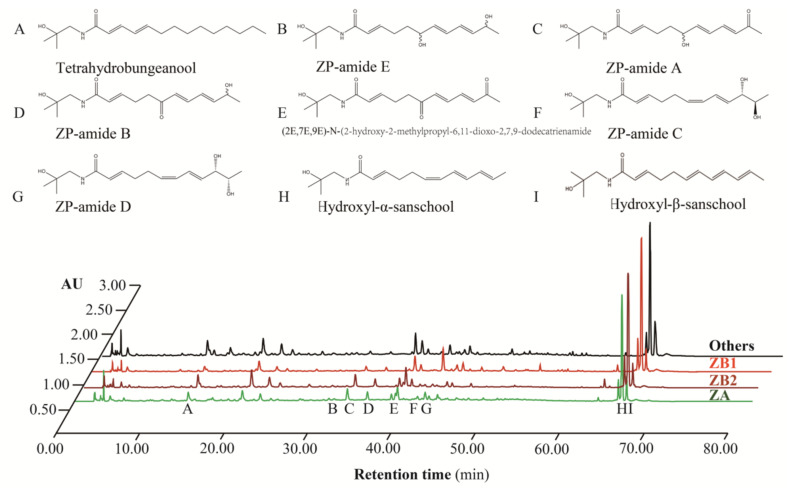
The relative abundance of alkylamides in different prickly ash species.

Determining qualitative and quantitative concentrations of alkylamides in prickly ash pericarps was carried out according to methods outlined in our previous research [27]. Alkylamide compositions in prickly ash pericarps were detected using 10 μL of injected volume and a flow rate of 1.0 mL/min at 30 °C in a UPLC system (Waters, Milford, MA, USA). The mobile phase was composed of solvent A (99.99% acetonitrile) and solvent B (1% methanoic acid) using the following program with a total time of 80 min: solvent A linearly increased from 5% to 14% during the first 25 min, to 25% by 45 min, to 40% after 55 min, and to 60% after 65 min. Then, solvent A was linearly reduced to 5% after 1 min, and it remained at 5% for the remaining 14 min of the program. Alkylamide profiling in the samples was identified by comparing the retention time with each authentic standard (Figure 2). The standards were homemade (purity ≥ 95%) and commercial (HPLC ≥ 98%, Shanghai yuanye Bio-Technology Co., Ltd. Shanghai, China). Quantification was performed using the external standard method. The results are presented as milligrams of each compound per g of dry pericarps (mg/g).

AU represents absorbance unit; the peaks without labels indicate non alkylamide components; ZP in alkylamides stands for *Z. piperitum*; ZA represents *Z. armatum*; ZB1 samples are derived from *Z. bungeanum* from Hancheng; ZB2 samples are from *Z. bungeanum* from Fengxian; Others refers to the rest of the samples that have red pericarps, but do not derive from *Z. bungeanum*.

### 2.4. Data Analyses

Univariate statistics were used to assess each variable independently with Tukey’s multiple comparisons (*p* < 0.01) in IBM SPSS Statistics 20.0 software. The data were presented in the form of Z-scores transformed before chemometric analyses if the values did not conform to a normally distributed population (Tables S1–S4). The *p*-value for the Kolmogorov–Smirnov normality test was used to assess whether the alkylamide data conformed to a normal distribution (Figure S1). Box plot, principal component analysis (PCA), and discriminant analysis (DA) were conducted using OriginPro 2018C (Originlab, Northampton, MA, USA). A cluster heat map (CHM) was performed using TBtools v1.0891 software [28]. Orthogonal partial least squares discriminant analysis (OPLS-DA) was carried out using http://www.omicshare.com/tools/Home/Soft/getsoft/type/index (1 July 2020). Moreover, the Canoco 5.0 program was used to conduct RDA.

## 3. Results

### 3.1. Alkylamide Profiling in Pericarps from Different Prickly Ash Groups

**Figure 3:**
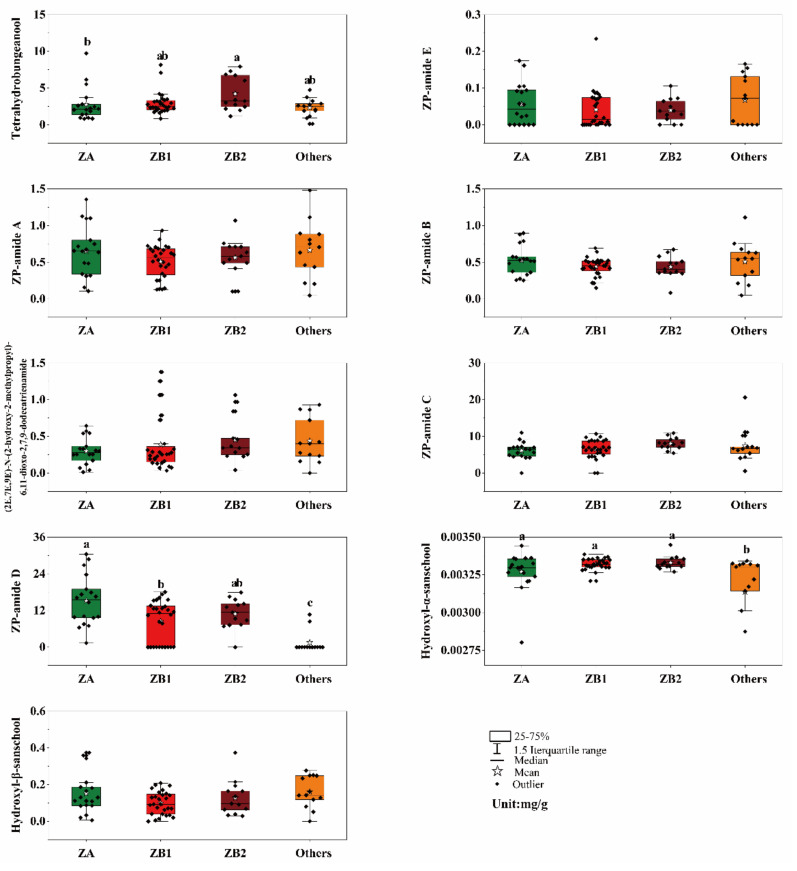
Alkylamide profiling in different prickly ash pericarps. The letters (a, b, c, and ab) above the histogram indicate significant differences among the prickly ash groups using the Student–Newman–Keuls test; the same letters (a or ab) and no letters above the histogram indicate no significant differences among different prickly ash groups (*p* < 0.01); the letter “a” indicates the highest content of the compound for the group; ZA represents green pericarps derived from the species of *Zanthoxylum armatum*; ZB1 represents red pericarps derived from the species of *Z. bungeanum* from Hancheng; ZB2 represents red pericarps from Fengxian; and Others includes the rest of the samples that have red pericarps (excluding *Z. bungeanum*).

A total of nine alkylamides were detected in most of the prickly ash pericarp samples, and alkylamide compositions in the prickly ash pericarps from different plantations were diverse (Figure 3). The most common components of the alkylamides in the prickly ash pericarps were ZP-amide C (Compound F, 0–20.64 mg/g) and ZP-amide D (Compound G, 0–30.43 mg/g). The contents of tetrahydrobungeanool (Compound A, 0.12–9.72 mg/g), Compound G, and Compound H (0.0021–0.0034 mg/g) in the pericarps demonstrated significant differences among the four groups, while the differences of ZP-amide E (Compound B, 0–0.23 mg/g), ZP-amide A (Compound C, 0.05–1.48 mg/g), ZP-amide B (Compound D, 0.05–1.11 mg/g), (2E,7E,9E)-N-(2-hydroxy-2-methylpropyl)-6,11-dioxo-2,7,9-dodecatrienamide (Compound E, 0–1.38 mg/g), Compound F, and Compound I (0–0.37 mg/g) were not significant (*p* < 0.01). The content of Compound A was the highest in the ZB2 pericarps, and the content of Compound G was the highest in the ZA pericarps. The content of Compound H was the lowest in the other pericarps.

### 3.2. Chemometric Analyses for Prickly Ash Pericarps Based on Alkylamide Profiling

The composition of alkylamides in prickly ash pericarps varied among different groups and plantations. To determine the important differential components between pericarps from different groups and determine the key environmental factors that caused the alkylamide variations, several chemometric analysis methods were conducted based on alkylamide profiling of prickly ash pericarps.

#### 3.2.1. Cluster Heat Map (CHM)

**Figure 4:**
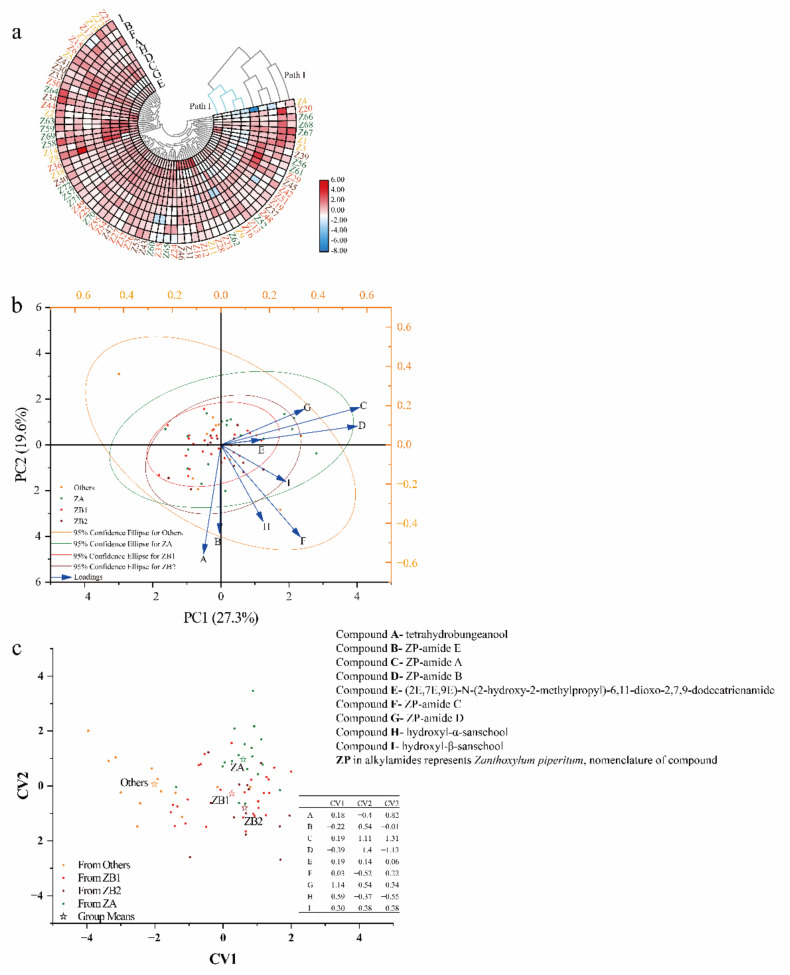
Geographical differentiation of prickly ash pericarps from 72 plantations based on alkylamide profiling of pericarps: (**a**) cluster heat map, (**b**) loading plot and score for the first two principal components, and (**c**) discriminant analysis. PC indicates principal component; CV stands for canonical variable; ZA (*n* = 18) represents green pericarps derived from the species of *Zanthoxylum armatum*; ZB1 (*n* = 28) represents red pericarps derived from the species of *Z. bungeanum* from Hancheng; ZB2 (*n* = 13) represents red pericarps from Fengxian; and Others (*n* = 13) represents the rest of the samples that have red pericarps, and is a mixture of species excluding *Z. bungeanum*.

First, CHM, an unsupervised pattern recognition method, was used to classify the 72 pericarp samples (Figure 4a). In addition, the differences and similarities in alkylamide content in the pericarps from different plantations were also observed. Compounds C, D, E, and G clustered into one group, while Compounds A, B, F, H, and I formed another group. All samples were put into one of three groups: the first group contained only Z4 (Laishui, Hebei, China), the second group contained 22 samples, and the third group contained most of the samples. The clusters of samples from different plantations did not agree with the results based on ITS2 and GBS simplified genome sequencing.

#### 3.2.2. Principal Component Analysis (PCA)

PCA, another unsupervised pattern recognition method, was used to better understand the chemometric characteristics of different pericarps (Figure 4b). PCA reflects the importance of each variable in relation to the total variation on each axis; furthermore, it reveals the distribution states of different samples [29,30]. The sample distribution characteristics of prickly ash pericarps from 72 plantations are shown in Figure 4a. When eigenvalue roots were greater than one, the principal components (PCs) were important. Four PCs were generated from the original data, accounting for 71.48% of the variation (27.35%, 19.61%, 12.63%, and 11.89%), and 29.52% of the information was lost. Nine alkylamides contributed to two principal components, and Compounds A, B, C, D, F, and H were far from the origin of coordinates. Compounds C and D contributed more to the variation of PC1, while Compounds A, B, F, and H contributed more to the variation of PC2. Moreover, some of the samples from different plantations and even from different genotypes overlapped.

#### 3.2.3. Discriminant Analysis (DA)

DA, a supervised analysis, confirmed a discrimination model to classify the pericarp samples (Figure 4c). Four discriminant groups (ZA, ZB1, ZB2, and Others) were generated as the train groups, and nine alkylamides were chosen as the variables before running the program (canonical discriminant analysis system). The prior probability was selected equally, and the discrimination function was selected linearly. For the discrimination function, Compound G in the pericarps contributed the most to the first canonical variable (CV), while Compound C and Compound D contributed the most to CV2 and CV3. The discrimination was as follows: nine alkylamides were substituted into three equations, and the unknown samples were compared to the means of the CV obtained from the model, CV1 (0.59 for ZA, 0.26 for ZB1, 0.64 for ZB2, and −2.02 for Others), CV2 (0.96 for ZA, −0.28 for ZB1, −0.80 for ZB2, and 0.06 for Others), and CV3 (0.17 for ZA, −0.47 for ZB1, 0.65 for ZB2, and 0.14 for Others); then, the samples were classified into groups. An error rate of 32.91% (27.78% for ZA, 50.00% for ZB1, 38.46% for ZB2, and 15.38% for Others) was generated from the discrimination functions using cross-validation.

#### 3.2.4. Orthogonal Partial Least Squares Discriminant Analysis (OPLS-DA)

Since alkylamides in the pericarps varied among the groups (ZA, ZB1, ZB2, and Others), OPLS-DA was used to determine the important differential indicators to distinguish each group. The explanatory rates of the model for ZA vs. ZB2, ZA vs. Others, ZB1 vs. Others, and ZB2 vs. Others were more than 0.4 based on R^2^X and R^2^Y; these groupings were acceptable. The predictive abilities of the model for ZA vs. Others and ZB2 vs. Others were acceptable. The verification results confirmed that the discrimination of Others was more accurate compared to the other groups (Table 1). The variables important in projection (VIP) varied in distinguishing different species. The VIP values of Compounds C, D, E, G, and I between ZA and ZB1, Compounds A, E, and F between ZA and ZB2, Compounds G and H between ZA and Others, Compounds A and F between ZB1 and ZB2, Compounds G, H, and I between ZB1 and Others, and Compounds A, G, and H between ZB2 and Others were more than one.

### 3.3. The Influences of Environmental Factors on Alkylamide Profiling

**Figure 5:**
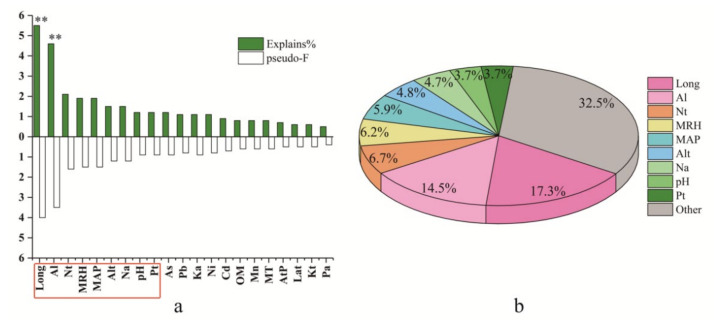
The effect of environmental factors on alkylamide variation for prickly ash pericarps: (**a**) the pseudo-F of each environmental factor and (**b**) key environmental factors. The symbol “**” represents the factor is significant; Long represents longitude; Al represents aluminum content in the soil; N_t_ represents total nitrogen content in the soil; MRH represents the mean relative humidity; N_a_ represents available nitrogen content in the soil; As represents arsenic content in the soil; pH represents power of hydrogen; MAP represents mean annual precipitation; Pb represents lead content in the soil; K_a_ represents available potassium content in the soil; P_t_ represents total phosphorus content in the soil; Ni represents nickel content in the soil; OM represents organic matter content in the soil; MT represents mean temperature; Alt represents altitude; Mn represents manganese content in the soil; Cd represents cadmium content in the soil; AtP represents atmospheric pressure; Lat represents latitude; K_t_ represents total potassium content in the soil; and P_a_ represents available phosphorus content in the soil.

The differences in alkylamide composition in prickly ash pericarps were identified for different groups and plantations. Consequently, the synergistic effect of genotype and environmental factors that affect alkylamide profiling need to be explored. The filtering of environmental variables was done using the procedure of interactive forward selection. The influence of each environmental factor on alkylamide variations is shown in Figure 5. Of these environmental variables, Long, Al, N_t_, MRH, MAP, Alt, N_a_, pH, and P_t_ were the key influence factors that caused alkylamide variations in prickly ash pericarps, because they had higher explanatory abilities (1.2–5.5%) and higher values of pseudo-F (0.9–4). Moreover, these key influence factors had higher contribution rates (3.7–17.3%), while the total contribution rate of the remaining factors was 32.5%. Of these influence factors, Long (pseudo-F = 4.0, *p* = 0.002) and Al (pseudo-F = 3.5, *p* = 0.006) were the most important key factors that influenced the alkylamide profiling of prickly ash pericarps.

**Figure 6:**
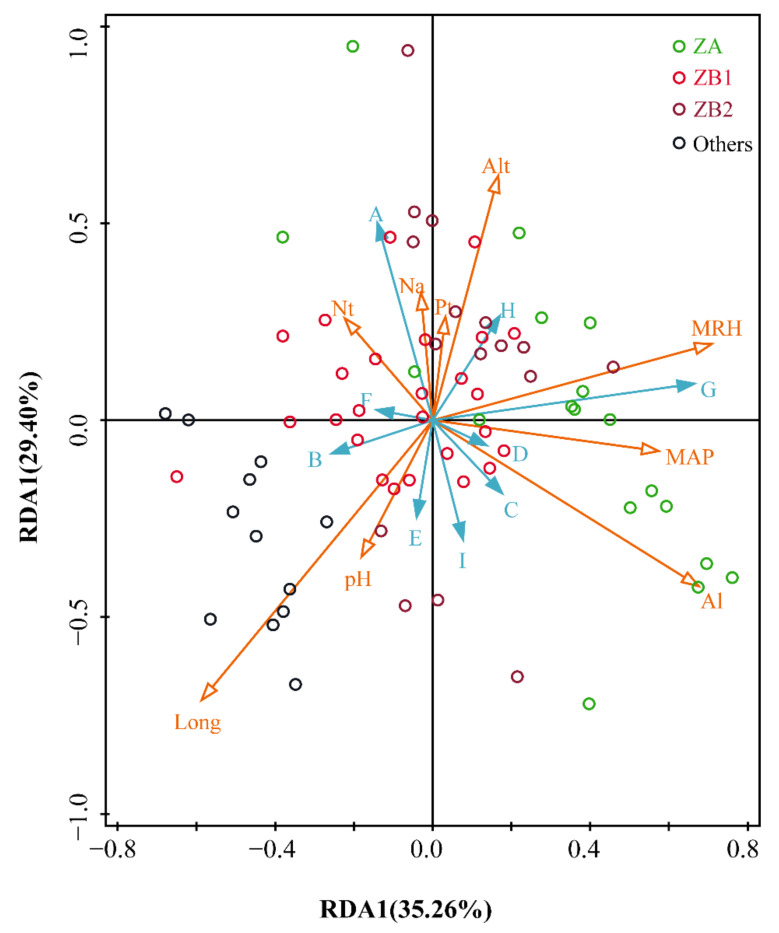
The relationships between environmental factors and alkylamide profiling in prickly ash pericarps.

As shown in Figure 6, 64.66% of the total variation in alkylamide compositions in the prickly ash pericarps (the explained portions of the first two RDA axes were 35.26% and 29.40%, respectively) were explained by environmental variables. The degree of influence of environmental factors is expressed by the arrow lengths in the figure: longer arrows represent greater influence on alkylamide variations. Long and Al have longer arrows compared to the other factors, indicating that they significantly influenced the alkylamide compositions of the prickly ash pericarps in the model. Moreover, the relationships between the samples are represented by the distances between two sites, and relationships between two variables are represented by the cosine values of two arrows [29]. The distributions of the sites were random and irregular. For Long and Al, Long had a positive effect on Compound B and Compound E, and had a negative effect on Compound H in the prickly ash pericarps, while Al in the soil had a positive effect on Compound C and Compound D, and a negative effect on Compound F in the pericarps.

RDA represents redundancy analysis; ZP in alkylamides represents *Zanthoxylum piperitum*; ZA (*n* = 18) represents green pericarps derived from *Zanthoxylum armatum*; ZB1 (*n* = 28) represents red pericarps derived from *Z. bungeanum* from Hancheng; ZB2 (*n* = 13) represents red pericarps from Fengxian; Others (*n* = 13) represents the rest of the samples that have red pericarps, excluding *Z. bungeanum*; A represents tetrahydrobungeanool; B represents ZP-amide E; C represents ZP-amide A; D represents ZP-amide B; E represents (2E,7E,9E)-N-(2-hydroxy-2-methylpropyl)-6,11-dioxo-2,7,9-dodecatrienamide; F represents ZP-amide C; G represents ZP-amide D; H represents hydroxyl-α-sanschool; I represents hydroxyl-β-sanschool; Long represents longitude; Al represents aluminum content in the soil; N_t_ represents total nitrogen content in the soil; MRH represents mean relative humidity; N_a_ represents available nitrogen content in the soil; As represents arsenic content in the soil; pH represents power of hydrogen; MAP represents mean annual precipitation; and Pb represents lead content in the soil.

## 4. Discussion

The different contents and components of alkylamides in prickly ash pericarps contributed to diverse numb sensations when the pericarps were used as spices and functional food [19]. A total of nine alkylamides were detected in the prickly ash pericarp samples, and alkylamide compositions in the prickly ash pericarps from different plantations were diverse. Genotype and environmental variation affected substance synthesis in the plants, and these substances often increased under external stresses [30]. Moreover, the differences among the components in *Zanthoxylum* plants were caused by the sample harvest time and extraction method [31,32]. The pericarps of prickly ash were harvested in the period of commodity maturity when some pericarps in the trees had cracked, and the sampling pericarps were dried and extracted under the same treatment conditions. Thus, the contents and component variations of alkylamides in prickly ash pericarps were influenced by prickly ash species and the environmental conditions of the plantations. Prickly ash pericarps generally always had Compound H, and this compound contributed the most to the numb flavor [10,13,19,20]. The presence of Compound H in the pericarps of the Others group was significantly low (the mean values for ZA, ZB1, and ZB2 were all 0.0033 mg/g, while the mean value of Others was 0.0031 mg/g), indicating that the pericarps of these species were not appealing materials to extract Compound H. Hence, ZA and ZB species, which contain more Compound H compared to Others, are widely cultivated and used as spices in China.

ZA, ZB, and some other *Zanthoxylum* species are botanically related, and they are often confused due to their similarities in morphological characteristics. Moreover, the overlapped distribution of some prickly ash species and inconsistent nomenclature of the species cultivated in different areas make distinguishing the pericarps more difficult. Chemical profiling in plants coupled with chemometrics has been widely used to identify potential indicators to distinguish and classify samples obtained from different species and from different origins [33,34,35]. Therefore, the chemometric analyses based on alkylamides in prickly ash pericarps were feasible and necessary. Five chemometric methods (CHM, PCA, DA, OPLS-DA, and RDA) were used to classify the prickly ash samples, to determine the key differences among pericarps from different prickly ash groups, and to determine the key environmental factors that caused the alkylamide variations. The alkylamides in the same group shared a similar synthesis and accumulation pathway of the alkylamides in the pericarps for the CHM model [36]. Compounds C, D, E, and G shared a similar synthesis and accumulation pathway in the pericarps, while Compounds A, B, F, H, and I shared another synthesis and accumulation pathway. For the PCA method, the variables with high negative or positive component loadings in PCA contributed more to the variation in each axis. Compound C and Compound D contributed more to the variation of PC1, while Compounds A, B, F, and H contributed more to the variation of PC2. CHM and PCA were unsupervised linear models [37,38]. The classification results did not agree with the previously determined groups based on the results of ITS2 and GBS simplified genome sequencing [7,11]. Therefore, the environmental factors in which the species are grown must have affected the synthesis of alkylamides in pericarps. The influence of intensity and the explanatory ability of each indicator on the discrimination of each group can be measured using VIP values. The indicators included in the OPLS-DA were important when the value of VIP was no less than one [39]. Compound B contributed less in terms of categorizing the samples, while Compound G was an important differential indicator to distinguish prickly ash species, especially for distinguishing Others from the rest of the species. DA provided an approach to distinguish different prickly ash groups, and indicated that the ZB1 and ZB2 species could be incorrectly categorized. ZB1 and ZB2 are widely planted in China, and botanically, ZB1, ZB2, and some Others species are often confused because of the similarity in morphological characteristics, especially for the ZB1 and ZB2 species; they even have the same genotype and similar red pericarps [11]. The species included in Others are mainly cultivated in the Shandong and Hebei provinces, but ZB1 is more widely cultivated. The prices of ZB1 and Others are inexpensive, and other more expensive species could be adulterated with ZB1 and Others, especially in pericarp powders. Thus, the important differential indicators may help to protect expensive products from possible fraud by verifying that samples comply with the actual label of the products.

Intraspecific genetic relationships from different plantations based on ITS2 marker data of the ZB species fell under a single cluster, but pericarps of these species have their own unique chemical compositions [9,13]. The environments of the plantations and molecular regulation of the plants affected the alkylamide compositions in prickly ash pericarps. The pericarps presented different chemical profiles for different cultivars grown under the same conditions [36] and for the same cultivar grown in different regions [9]. This study highlights more detailed relationships between influence factors and alkylamide variations compared to previous studies. Compounds A, B, C, D, F, and H contributed the most to the variations in the alkylamide compositions of the prickly ash pericarps. Long and Al were the main factors that affected the alkylamide compositions in prickly ash pericarps, and the influences of these two factors on phytochemical profiling have also been reported in other plants [40,41].

## 5. Conclusions

In this study, nine alkylamide components in prickly ash pericarps were identified. Compound F and Compound G were the most common components of the prickly ash pericarps. Alkylamide profiling of the prickly ash pericarps was particularly affected by the species of plants and by Long and Al in the soil. Compound G was an important differential indicator in distinguishing prickly ash species, especially for distinguishing Others from the rest of the species. The results of this study provided a powerful tool to classify prickly ash species based on pericarp alkylamide compositions, and they illuminate the relationships between the composition and content of alkylamides and environmental factors. Moreover, this study helps to protect pericarps from possible fraud by determining if samples comply with the product profiling. It can also help improve the quality of pericarps by introducing better prickly ash cultivars and changing the cultivated environments.

## Figures and Tables

**Table 1:** Variables important in projection and verification by permutation test between different prickly ash groups.

Indicator	ZA vs. ZB1	ZA vs. ZB2	ZA vs. Others	ZB1 vs. ZB2	ZB1 vs. Others	ZB2 vs. Others
Tetrahydrobungeanool	0.21	1.72	0.18	2.10	0.70	1.32
ZP-amide E	0.82	0.47	0.59	0.01	0.75	0.81
ZP-amide A	1.16	0.69	0.12	0.13	0.76	0.67
ZP-amide B	1.09	0.55	0.05	0.09	0.49	0.66
(2E,7E,9E)-N-(2-hydroxy-2-methylpropyl)-6,11-dioxo-2,7,9-dodecatrienamide	1.02	1.16	0.78	0.56	0.13	0.06
ZP-amide C	0.26	1.57	0.99	1.67	0.55	0.14
ZP-amide D	1.72	0.84	2.38	0.77	1.66	1.79
hydroxyl-α-sanschool	0.49	0.66	1.15	0.27	1.68	1.45
hydroxyl-β-sanschool	1.21	0.31	0.16	0.89	1.10	0.64
R^2^X	0.47	0.49	0.41	0.37	0.42	0.43
R^2^Y	0.36	0.51	0.74	0.24	0.51	0.68
Q^2^Y	0.12	0.26	0.66	0.02	0.36	0.44

R^2^X represents the explanatory rate for the X matrix in the model; R^2^Y represents the explanatory rate for the Y matrix in the model; Q^2^Y represents the predictive ability of the model. In theory, the model is better when R^2^X, R^2^Y, and Q^2^Y are closer to one; usually, the model is better if R^2^X, R^2^Y, and Q^2^Y are higher than 0.5, and it is acceptable if R^2^X, R^2^Y, and Q^2^Y are higher than 0.4. ZP in alkylamides represents *Zanthoxylum piperitum*; ZA (*n* = 18) represents green pericarps derived from *Zanthoxylum armatum*; ZB1 (*n* = 28) represents red pericarps derived from *Z. bungeanum* from Hancheng; ZB2 (*n* = 13) represents red pericarps from Fengxian; Others (*n* = 13) includes the rest of the samples that have red pericarps, and is a mixture of species excluding *Z. bungeanum*.

## Supplementary Materials

The following are available online at https://www.mdpi.com/article/10.3390/foods10040866/s1, Table S1: Raw data and transformed (Z-Score) values of location and climate for the 72 prickly ash pericarp samples. ZA, samples with green pericarps (*n* = 18) derived from *Z, armatum*; ZB1, samples with red pericarps from Hancheng (*n* = 28) derived from *Z. bungeanum*; ZB2, samples with red pericarps from Fengxian (*n* = 13) derived from *Z. bungeanum*; Others, samples with red pericarps, a class of several species (*n* = 13), excluding *Z. bungeanum*. Table S2: Raw data and transformed (Z-Score) values of soil characteristics in different prickly ash plantations. ZA, samples with green pericarps (*n* = 18) derived from *Z, armatum*; ZB1, samples with red pericarps from Hancheng (*n* = 28) derived from *Z. bungeanum*; ZB2, samples with red pericarps from Fengxian (*n* = 13) derived from *Z. bungeanum*; Others, samples with red pericarps, a class of several species (*n* = 13), excluding *Z. bungeanum*. Table S3: Raw data and transformed (Z-Score) values of potentially toxic elements in prickly ash soils. ZA, samples with green pericarps (*n* = 18) derived from *Z, armatum*; ZB1, samples with red pericarps from Hancheng (*n* = 28) derived from *Z. bungeanum*; ZB2, samples with red pericarps from Fengxian (*n* = 13) derived from *Z. bungeanum*; Others, samples with red pericarps, a class of several species (*n* = 13), excluding *Z. bungeanum*. Table S4: Transformed (Z-Score) values of alkylamide data in different prickly ash pericarps. ZA, samples with green pericarps (*n* = 18) derived from *Z, armatum*; ZB1, samples with red pericarps from Hancheng (*n* = 28) derived from *Z. bungeanum*; ZB2, samples with red pericarps from Fengxian (*n* = 13) derived from *Z. bungeanum*; Others, samples with red pericarps, a class of several species (*n* = 13), excluding *Z. bungeanum*; ZP in alkylamides represents *Z. piperitum*. Figure S1: Mean, median, standard deviation (SD), variance (CV), skewness coefficient, and *p*-value for the Kolmogorov–Smirnov normality test of alkylamides (mg/g dry pericarps).

## List of Abbreviations

ZA refers to samples that have green pericarps (*n* = 18) derived from *Z, armatum*; ZB1 refers to samples that have red pericarps from Hancheng (*n* = 28) derived from *Z. bungeanum*; ZB2 refers to samples that have red pericarps from Fengxian (*n* = 13) derived from *Z. bungeanum*; Others refers to samples that have red pericarps and is a class of several species (*n* = 13), excluding *Z. bungeanum*; ZB refers to *Z. bungeanum*; ZP in alkylamides refers to *Zanthoxylum piperitum*; Compound A is tetrahydrobungeanool; Compound B is ZP-amide E; Compound C is ZP-amide A; Compound D is ZP-amide B; Compound E is (2E,7E,9E)-N-(2-hydroxy-2-methylpropyl)-6,11-dioxo-2,7,9-dodecatrienamide; Compound F is ZP-amide C; Compound G is ZP-amide D; Compound H is hydroxyl-α-sanschool; Compound I is hydroxyl-β-sanschool; Long means longitude; Al refers to aluminum content in soil; N_t_ refers to total nitrogen content in soil; MRH is the mean relative humidity; N_a_ is available nitrogen content in soil; As refers to arsenic content in soil; pH means power of hydrogen; MAP is the mean annual precipitation; Pb refers to lead content in soil; K_a_ is the available potassium content in soil; P_t_ is the total phosphorus content in soil; Ni is the nickel content in soil; OM refers to organic matter content in soil; MT is the mean temperature; Alt means altitude; Mn is the manganese content in soil; Cd refers to cadmium content in soil; AtP is the atmospheric pressure; Lat means latitude; K_t_ is the total potassium content in soil; P_a_ is the available phosphorus content in soil; AU means absorbance unit; CHM refers to cluster heat map; PCA refers to principal component analysis; PC is principal component; DA is discriminant analysis; CV means canonical variable, OPLS-DA is orthogonal partial least squares discriminant analysis; VIP refers to variables important in projection; R^2^X is the explanatory rate for the X matrix in the model; R^2^Y is the explanatory rate for the Y matrix in the model; Q^2^Y is the predictive ability of the model; RDA means redundancy analysis; UPLC is ultra-performance liquid chromatography; HPLC is high-performance liquid chromatography.
