# Supplementary material for: Alkylamide Profiling of Pericarps Coupled with Chemometric Analysis to Distinguish Prickly Ash Pericarps
Source: Foods. 2021 Apr 15;10(4):866. doi: 10.3390/foods10040866 (PMC8071439; doi:10.3390/foods10040866)
Supplement: Supplementary file 1 [file foods-10-00866-s001.zip › Supplementary Figures.pdf]

Supplementary figures

Alkylamide Profiling of Pericarps Coupled with Chemometric Analysis to Distinguish Prickly Ash Pericarps

Yao Ma [1,2], Lu Tian [1,2], Xiaona Wang [1,2], Chen Huang [1], Mingjing Tian [1], and Anzhi Wei [1,2,*]

[1] College of Forestry, Northwest A&F University, Yangling 712100, China; mayao277000@nwafu.edu.cn (Y.M.); t1anlu@nwafu.edu.cn (L.T.); 17709590958@nwafu.edu.cn (X.W.); hc19990513@nwafu.edu.cn (C.H.); 15531971892@nwafu.edu.cn (M.T.); weianzhi@nwafu.edu.cn (A.W.)

[2] Research Centre for Engineering and Technology of *Zanthoxylum*, State Forestry Administration, Yangling 712100, China

* Correspondence: weianzhi@nwafu.edu.cn; Tel.: +86-029-8708-2211

Supplementary figures

Figure S1 Mean, median, standard deviation (SD), variance (CV), skewness coefficient, and *p* value for the Kolmogorov–Smirnov normality test of alkylamides (mg/g dry pericarps).

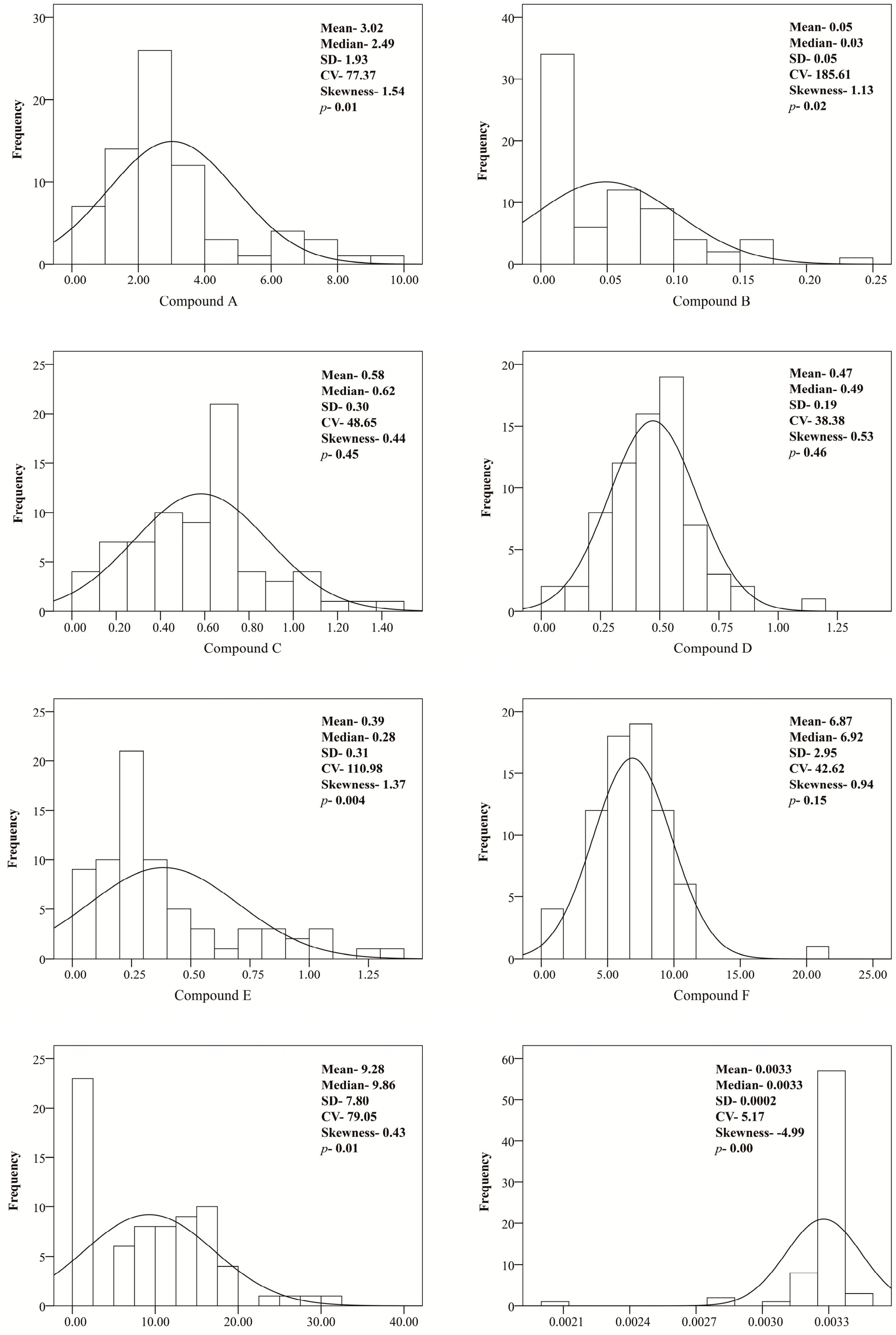

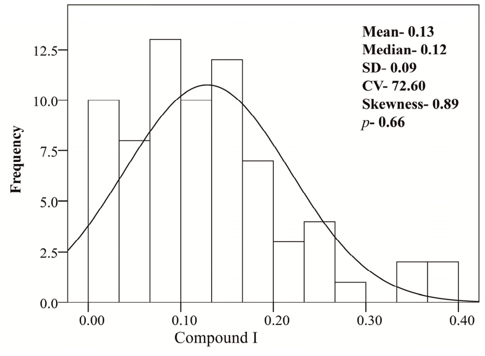

Compound **A-** tetrahydrobungeanool
Compound **B-** ZP-amide E
Compound **C-** ZP-amide A
Compound **D-** ZP-amide B
Compound **E-** (2E,7E,9E)-N-(2-hydroxy-2-methylpropyl)-6,11-dioxo-2,7,9-dodecatrienamide
Compound **F-** ZP-amide C
Compound **G-** ZP-amide D
Compound **H-** hydroxyl-α-sanschool
Compound **I-** hydroxyl-β-sanschool
ZP in alkylamides represents *Zanthoxylum piperitum*, nomenclature of compound

Figure S1 Mean, median, standard deviation (SD), variance (CV), skewness coefficient, and *p* value for the Kolmogorov–Smirnov normality test of alkylamides (mg/g dry pericarps).
