# Supplementary material for: Alkylamide Profiling of Pericarps Coupled with Chemometric Analysis to Distinguish Prickly Ash Pericarps
Source: Foods. 2021 Apr 15;10(4):866. doi: 10.3390/foods10040866 (PMC8071439; doi:10.3390/foods10040866)
Supplement: Supplementary file 1 [file foods-10-00866-s001.zip › Supplementary Tables.pdf]

Supplementary tables

Alkylamide Profiling of Pericarps Coupled with Chemometric Analysis to Distinguish Prickly Ash Pericarps

Yao Ma [1,2], Lu Tian [1,2], Xiaona Wang [1,2], Chen Huang [1], Mingjing Tian [1], and Anzhi Wei [1,2,*]

1 College of Forestry, Northwest A&F University, Yangling 712100, China; mayao277000@nwafu.edu.cn (Y.M.); t1anlu@nwafu.edu.cn (L.T.); 17709590958@nwafu.edu.cn (X.W.); hc19990513@nwafu.edu.cn (C.H.); 15531971892@nwafu.edu.cn (M.T.); weianzhi@nwafu.edu.cn (A.W.)

2 Research Centre for Engineering and Technology of *Zanthoxylum*, State Forestry Administration, Yangling 712100, China

* Correspondence: weianzhi@nwafu.edu.cn; Tel.: +86-029-8708-2211

Supplementary tables

Table S1 Raw data and transformed (Z-Score) values of location and climate for the 72 prickly ash pericarp samples.

ZA, samples with green pericarps (n=18) derived from group of *Z, armatum*; ZB1, samples with red pericarps from Hancheng (n=28) derived from group of *Z. bungeanum*; ZB2, samples with red pericarps from Fengxian (n=13) derived from *Z. bungeanum*; Others, samples with red pericarps, a class of several species (n=13), excluding *Z. bungeanum.*

Table S2 Raw data and transformed (Z-Score) values of soil characteristics in different prickly ash plantations.

ZA, samples with green pericarps (n=18) derived from group of *Z, armatum*; ZB1, samples with red pericarps from Hancheng (n=28) derived from group of *Z. bungeanum*; ZB2, samples with red pericarps from Fengxian (n=13) derived from group of *Z. bungeanum*; Others, samples with red pericarps, a class of several species (n=13), excluding *Z. bungeanum.*

Table S3 Raw data and transformed (Z-Score) values of potentially toxic elements in prickly ash soils.

ZA, samples with green pericarps (n=18) derived from group of *Z, armatum*; ZB1, samples with red pericarps from Hancheng (n=28) derived from group of *Z. bungeanum*; ZB2, samples with red pericarps from Fengxian (n=13) derived from group of *Z. bungeanum*; Others, samples with red pericarps, a class of several species (n=13), excluding *Z. bungeanum.*

Table S4 Transformed (Z-Score) values of alkylamides data in different prickly ash pericarps.

ZA, samples with green pericarps (n=18) derived from group of *Z, armatum*; ZB1, samples with red pericarps from Hancheng (n=28) derived from group of *Z. bungeanum*; ZB2, samples with red pericarps from Fengxian (n=13) derived from group of *Z. bungeanum*; Others, samples with red pericarps, a class of several species (n=13), excluding *Z. bungeanum*; ZP in alkylamides represents *Z. piperitum.*

Table S1 Raw data and transformed (Z-Score) values of location and climate for the 72 prickly ash pericarp samples.

Group	Code	Origin Province	County	Longitude	Latitude	Altitude	Atmospheric pressure	Mean temperature	Mean relative humidity	Mean annual precipitation
Others	Z1	Shandong	Yiyuan	118.33	36.03	210	981.7	12.5	62	689.5
Others	Z2	Shandong	Laicheng	117.66	36.20	192	990.2	13.3	62	695.3
Others	Z3	Shandong	Shanting	117.59	35.06	195	1008.0	14.4	66	820.2
Others	Z4	Hebei	Laishui	115.45	39.67	298	1013.2	12.2	62	531
Others	Z5	Hebei	Tangxian	114.78	38.86	191	1008.9	12.7	65	508.1
Others	Z7	Hebei	Shexian	113.66	36.55	433	961.8	12.8	61	535
Others	Z8	Hebei	Fuping	114.20	38.85	274	983.4	12.6	56	572.1
Others	Z9	Hebei	Pingshan	114.20	38.25	138	983.4	12.6	56	572.1
Others	Z10	Shanxi	Wutai	113.26	38.73	1032	892.4	7.0	59	503.7
Others	Z11	Shanxi	Xiaoyi	111.78	37.15	753	928.0	11.0	57	448.5
Others	Z14	Shanxi	Yangcheng	112.41	35.49	628	940.8	12.0	61	578.4
Others	Z22	Shaanxi	Huanglong	109.72	35.60	1138	893.8	9.0	63	566.2
Others	Z38	Gansu	Dongxiang	103.24	35.74	1807	759.5	5.6	62	534.9
ZB1	Z6	Hebei	Shexian	113.66	36.55	433	961.8	12.8	61	535
ZB1	Z12	Shanxi	Zaoning	110.66	35.80	835	907.1	10.5	59	515
ZB1	Z13	Shanxi	Pingshun	113.65	36.36	578	893.6	9.4	59	553
ZB1	Z15	Shanxi	Ruicheng	110.69	34.69	504	957.9	13.0	67	505.3
ZB1	Z16	Henan	Lingbao	110.59	34.50	692	961.7	13.5	66	600.5
ZB1	Z17	Henan	Hubin	111.19	34.77	358	969.1	14.4	61	549.6
ZB1	Z18	Henan	Linzhou	113.94	36.26	396	980.8	13.2	66	648.9
ZB1	Z19	Henan	Baofeng	112.90	33.93	212	1000.7	14.6	69	747.2

ZB1	Z20	Shaanxi	Yanchang	110.41	36.51	968	924.6	10.4	62	480.8
ZB1	Z21	Shaanxi	Yichuan	110.37	35.94	793	921.0	10.3	61	516
ZB1	Z23	Shaanxi	Hancheng	110.47	35.55	452	935.0	12.1	64	535.4
ZB1	Z24	Shaanxi	Heyang	110.32	35.30	549	935.0	12.1	64	535.4
ZB1	Z25	Shaanxi	Huazhou	109.72	34.43	741	975.3	13.7	71	577.5
ZB1	Z26	Shaanxi	Fuping	109.12	34.82	526	961.6	12.1	68	636.5
ZB1	Z27	Shaanxi	Yaozhou	109.03	34.94	820	934.5	12.7	62	543.4
ZB1	Z28	Shaanxi	Chunhua	108.66	34.75	737	902.0	10.6	67	585.7
ZB1	Z29	Shaanxi	Chunhua	108.66	34.75	737	902.0	10.6	67	585.7
ZB1	Z32	Shaanxi	Chencang	106.45	34.53	915	946.1	13.5	66	645.8
ZB1	Z35	Qinghai	Xunhua	102.49	35.85	1870	812.6	9.0	54	271.8
ZB1	Z36	Gansu	Jishishan	103.08	35.68	1841	808.1	7.3	67	501.2
ZB1	Z42	Gansu	Gangu	105.34	34.73	1300	874.0	10.7	68	437.3
ZB1	Z44	Gansu	Xihe	105.29	33.74	1086	842.4	9.1	73	527.9
ZB1	Z48	Sichuan	Hanyuan	102.64	29.35	939	923.9	17.8	69	755.6
ZB1	Z49	Sichuan	Mianning	102.19	28.29	1654	820.6	14.1	70	1106.7
ZB1	Z50	Sichuan	Yiyuan	101.51	27.42	2563	999.0	12.3	60	828.6
ZB1	Z51	Yunnan	Yongping	99.54	25.46	1667	999.0	15.8	75	951.7
ZB1	Z54	Guizhou	Zhenfeng	105.63	25.61	1224	894.5	16.6	79	1318.3
ZB1	Z55	Guizhou	Qixingguan	105.31	27.30	1511	848.7	13.0	81	866.1
ZB2	Z30	Shaanxi	Yangling	108.01	34.30	528	904.8	12.1	68	636.5
ZB2	Z31	Shaanxi	Chencang	106.70	106.70	1050	946.1	13.5	66	645.8
ZB2	Z33	Shaanxi	Taibai	107.12	33.99	1401	846.5	8.1	70	712.7
ZB2	Z34	Shaanxi	Fengxian	106.65	33.97	1061	904.8	12.1	68	636.5
ZB2	Z37	Gansu	Yongjing	103.29	35.96	1633	835.3	9.7	59	273.8
ZB2	Z39	Gansu	Linxia	103.15	35.75	1789	808.1	7.3	67	501.2

ZB2	Z40	Gansu	Jingning	105.73	35.52	1668	834.4	7.8	66	414
ZB2	Z41	Gansu	Qin'an	105.80	35.06	1404	880.1	10.9	66	436.3
ZB2	Z43	Gansu	Lixian	105.05	33.79	1473	860.3	10.3	71	470.7
ZB2	Z45	Gansu	Wudu	110.47	35.55	452	893.6	14.9	58	460.8
ZB2	Z46	Sichuan	Songpan	103.60	32.66	2865	720.9	6.3	63	708.3
ZB2	Z47	Sichuan	Maoxian	103.85	31.68	1602	840.6	11.2	74	462.3
ZB2	Z53	Yunnan	Ludian	103.23	27.31	1356	999.0	12.2	76	852.8
ZA	Z52	Yunnan	Qiaojiaxian	103.15	27.35	1733	916.2	20.8	60	838.2
ZA	Z56	Sichuan	Hanyuan	102.39	29.68	1641	923.9	17.8	69	755.6
ZA	Z57	Sichuan	Wusheng	106.30	30.35	288	977.5	17.5	84	1051.1
ZA	Z58	Sichuan	Daxian	107.25	31.19	478	974.4	17.2	80	1205.1
ZA	Z59	Sichuan	Pengxi	105.71	30.76	366	969.4	16.8	81	929.1
ZA	Z60	Sichuan	Lezhi	104.95	30.21	424	972.7	17.3	81	859.8
ZA	Z61	Sichuan	Jinyang	103.25	27.70	1288	791.6	15.9	70	795
ZA	Z62	Chongqing	Jiangjin	106.12	29.06	250	984.0	18.3	81	1000.9
ZA	Z63	Chongqing	Qianjiang	106.65	29.03	235	945.6	15.7	79	1172.8
ZA	Z64	Chongqing	Fengdu	107.71	30.22	397	980.3	18.3	80	1018.7
ZA	Z65	Chongqing	Fengdu	107.71	30.22	397	980.3	18.3	80	1018.7
ZA	Z66	Yunan	Yongshan	103.63	28.23	844	804.8	16.6	74	684.8
ZA	Z67	Yunan	Ludian	103.23	27.31	1356	999.0	12.2	76	852.8
ZA	Z68	Yunan	Qiaojiaxian	102.98	27.36	1324	916.2	20.8	60	838.2
ZA	Z69	Guizhou	Qixingguan	105.31	27.30	1511	848.7	13.0	81	866.1
ZA	Z70	Guizhou	Guanling	105.66	25.68	591	887.0	16.4	79	1327.6
ZA	Z71	Guizhou	Zhenfeng	105.63	25.61	1224	894.5	16.6	79	1318.3
ZA	Z72	Jiangxi	Xiushui	114.16	29.06	151	998.9	16.8	79	1601.8

Transformed

(Z-Score)										
Others	Z1	Shandong	Yiyuan	2.34	0.21	-1.19	0.93	-0.15	-0.77	-0.06
Others	Z2	Shandong	Laicheng	2.19	0.23	-1.22	1.06	0.09	-0.77	-0.04
Others	Z3	Shandong	Shanting	2.17	0.11	-1.21	1.32	0.42	-0.25	0.44
Others	Z4	Hebei	Laishui	1.69	0.60	-1.04	1.40	-0.24	-0.77	-0.66
Others	Z5	Hebei	Tangxian	1.54	0.51	-1.22	1.34	-0.09	-0.38	-0.75
Others	Z7	Hebei	Shexian	1.28	0.27	-0.82	0.64	-0.06	-0.90	-0.65
Others	Z8	Hebei	Fuping	1.41	0.51	-1.08	0.96	-0.12	-1.55	-0.51
Others	Z9	Hebei	Pingshan	1.41	0.45	-1.31	0.96	-0.12	-1.55	-0.51
Others	Z10	Shanxi	Wutai	1.19	0.50	0.17	-0.40	-1.77	-1.16	-0.77
Others	Z11	Shanxi	Xiaoyi	0.86	0.33	-0.29	0.13	-0.59	-1.42	-0.98
Others	Z14	Shanxi	Yangcheng	1.00	0.16	-0.50	0.32	-0.29	-0.90	-0.48
Others	Z22	Shaanxi	Huanglong	0.39	0.17	0.34	-0.38	-1.18	-0.64	-0.53
Others	Z38	Gansu	Dongxiang	-1.07	0.18	1.45	-2.38	-2.19	-0.77	-0.65
ZB1	Z6	Hebei	Shexian	1.28	0.27	-0.82	0.64	-0.06	-0.90	-0.65
ZB1	Z12	Shanxi	Zaoning	0.61	0.19	-0.16	-0.18	-0.74	-1.16	-0.72
ZB1	Z13	Shanxi	Pingshun	1.28	0.25	-0.58	-0.38	-1.06	-1.16	-0.58
ZB1	Z15	Shanxi	Ruicheng	0.61	0.07	-0.70	0.58	0.00	-0.12	-0.76
ZB1	Z16	Henan	Lingbao	0.59	0.05	-0.39	0.63	0.15	-0.25	-0.40
ZB1	Z17	Henan	Hubin	0.73	0.08	-0.94	0.74	0.42	-0.90	-0.59
ZB1	Z18	Henan	Linzhou	1.35	0.24	-0.88	0.92	0.06	-0.25	-0.21
ZB1	Z19	Henan	Baofeng	1.11	-0.01	-1.19	1.21	0.48	0.13	0.16
ZB1	Z20	Shaanxi	Yanchang	0.55	0.26	0.06	0.08	-0.77	-0.77	-0.85
ZB1	Z21	Shaanxi	Yichuan	0.54	0.20	-0.23	0.03	-0.80	-0.90	-0.72
ZB1	Z23	Shaanxi	Hancheng	0.56	0.16	-0.79	0.24	-0.26	-0.51	-0.65
ZB1	Z24	Shaanxi	Heyang	0.53	0.14	-0.63	0.24	-0.26	-0.51	-0.65

ZB1	Z25	Shaanxi	Huazhou	0.39	0.04	-0.31	0.84	0.21	0.39	-0.49
ZB1	Z26	Shaanxi	Fuping	0.26	0.09	-0.67	0.63	-0.26	0.01	-0.26
ZB1	Z27	Shaanxi	Yaozhou	0.24	0.10	-0.18	0.23	-0.09	-0.77	-0.62
ZB1	Z28	Shaanxi	Chunhua	0.15	0.08	-0.32	-0.26	-0.71	-0.12	-0.46
ZB1	Z29	Shaanxi	Chunhua	0.15	0.08	-0.32	-0.26	-0.71	-0.12	-0.46
ZB1	Z32	Shaanxi	Chencang	-0.34	0.06	-0.02	0.40	0.15	-0.25	-0.23
ZB1	Z35	Qinghai	Xunhua	-1.24	0.19	1.55	-1.59	-1.18	-1.80	-1.65
ZB1	Z36	Gansu	Jishishan	-1.11	0.18	1.50	-1.65	-1.68	-0.12	-0.78
ZB1	Z42	Gansu	Gangu	-0.60	0.08	0.61	-0.67	-0.68	0.01	-1.02
ZB1	Z44	Gansu	Xihe	-0.61	-0.03	0.26	-1.14	-1.15	0.65	-0.68
ZB1	Z48	Sichuan	Hanyuan	-1.21	-0.49	0.01	0.07	1.42	0.13	0.19
ZB1	Z49	Sichuan	Mianning	-1.31	-0.60	1.20	-1.47	0.33	0.26	1.53
ZB1	Z50	Sichuan	Yiyuan	-1.46	-0.69	2.70	1.19	-0.21	-1.03	0.47
ZB1	Z51	Yunnan	Yongping	-1.91	-0.90	1.22	1.19	0.83	0.91	0.94
ZB1	Z54	Guizhou	Zhenfeng	-0.53	-0.88	0.49	-0.37	1.07	1.43	2.34
ZB1	Z55	Guizhou	Qixingguan	-0.60	-0.71	0.96	-1.05	0.00	1.69	0.61
ZB2	Z30	Shaanxi	Yangling	0.01	0.03	-0.66	-0.21	-0.26	0.01	-0.26
ZB2	Z31	Shaanxi	Chencang	-0.29	7.66	0.20	0.40	0.15	-0.25	-0.23
ZB2	Z33	Shaanxi	Taibai	-0.19	0.00	0.78	-1.08	-1.45	0.26	0.03
ZB2	Z34	Shaanxi	Fengxian	-0.30	0.00	0.22	-0.21	-0.26	0.01	-0.26
ZB2	Z37	Gansu	Yongjing	-1.06	0.21	1.16	-1.25	-0.97	-1.16	-1.64
ZB2	Z39	Gansu	Linxia	-1.09	0.18	1.42	-1.65	-1.68	-0.12	-0.78
ZB2	Z40	Gansu	Jingning	-0.51	0.16	1.22	-1.26	-1.54	-0.25	-1.11
ZB2	Z41	Gansu	Qin'an	-0.49	0.11	0.78	-0.58	-0.62	-0.25	-1.02
ZB2	Z43	Gansu	Lixian	-0.66	-0.02	0.90	-0.88	-0.80	0.39	-0.89
ZB2	Z45	Gansu	Wudu	0.56	0.16	-0.79	-0.38	0.56	-1.29	-0.93

ZB2	Z46	Sichuan	Songpan	-0.99	-0.14	3.19	-2.95	-1.98	-0.64	0.01
ZB2	Z47	Sichuan	Maoxian	-0.93	-0.25	1.11	-1.17	-0.53	0.78	-0.93
ZB2	Z53	Yunnan	Ludian	-1.07	-0.71	0.70	1.19	-0.24	1.04	0.56
ZA	Z52	Yunnan	Qiaojiaxian	-1.09	-0.70	1.33	-0.04	2.31	-1.03	0.51
ZA	Z56	Sichuan	Hanyuan	-1.26	-0.46	1.17	0.07	1.42	0.13	0.19
ZA	Z57	Sichuan	Wusheng	-0.38	-0.39	-1.06	0.87	1.33	2.07	1.32
ZA	Z58	Sichuan	Daxian	-0.16	-0.30	-0.75	0.82	1.24	1.56	1.91
ZA	Z59	Sichuan	Pengxi	-0.51	-0.34	-0.93	0.75	1.13	1.69	0.85
ZA	Z60	Sichuan	Lezhi	-0.68	-0.40	-0.84	0.80	1.27	1.69	0.59
ZA	Z61	Sichuan	Jinyang	-1.07	-0.66	0.59	-1.90	0.86	0.26	0.34
ZA	Z62	Chongqing	Jiangjin	-0.42	-0.52	-1.12	0.97	1.57	1.69	1.13
ZA	Z63	Chongqing	Qianjiang	-0.30	-0.52	-1.15	0.39	0.80	1.43	1.78
ZA	Z64	Chongqing	Fengdu	-0.06	-0.40	-0.88	0.91	1.57	1.56	1.20
ZA	Z65	Chongqing	Fengdu	-0.06	-0.40	-0.88	0.91	1.57	1.56	1.20
ZA	Z66	Yunan	Yongshan	-0.98	-0.61	-0.14	-1.70	1.07	0.78	-0.08
ZA	Z67	Yunan	Ludian	-1.07	-0.71	0.70	1.19	-0.24	1.04	0.56
ZA	Z68	Yunan	Qiaojiaxian	-1.13	-0.70	0.65	-0.04	2.31	-1.03	0.51
ZA	Z69	Guizhou	Qixingguan	-0.60	-0.71	0.96	-1.05	0.00	1.69	0.61
ZA	Z70	Guizhou	Guanling	-0.52	-0.88	-0.56	-0.48	1.01	1.43	2.37
ZA	Z71	Guizhou	Zhenfeng	-0.53	-0.88	0.49	-0.37	1.07	1.43	2.34
ZA	Z72	Jiangxi	Xiushui	1.40	-0.52	-1.29	1.19	1.13	1.43	3.42

ZA, samples with green pericarps (n=18) derived from group of *Z, armatum*; ZB1, samples with red pericarps from Hancheng (n=28) derived from group of *Z. bungeanum*; ZB2, samples with red pericarps from Fengxian (n=13) derived from *Z. bungeanum*; Others, samples with red pericarps, a class of several species (n=13), excluding *Z. bungeanum.*

Table S2 Raw data and transformed (Z-Score) values of soil characteristics in different prickly ash plantations

Group	Code	Raw data								Transformed (Z-Score) values							
		pH	OM	N_t	P_t	K_t	N_a	P_a	K_a	pH	OM	N_t	P_t	K_t	N_a	P_a	K_a
Others	Z1	7.80	44.46	2.21	0.62	16.22	79.38	4.28	320.30	0.02	0.52	0.72	-0.72	0.49	0.06	-0.67	-0.30
Others	Z2	7.47	27.89	1.35	1.65	16.62	48.58	175.13	161.30	-0.34	-0.20	-0.18	0.68	0.60	-0.33	2.06	-1.01
Others	Z3	7.14	41.70	1.85	0.77	13.44	80.78	28.28	253.50	-0.70	0.40	0.35	-0.51	-0.22	0.07	-0.29	-0.60
Others	Z4	8.05	58.14	2.39	1.08	14.05	80.78	54.63	580.50	0.29	1.12	0.89	-0.10	-0.07	0.07	0.13	0.87
Others	Z5	8.13	48.45	1.94	1.25	12.72	84.98	55.88	822.00	0.38	0.70	0.44	0.13	-0.41	0.13	0.15	1.96
Others	Z7	8.28	26.88	1.09	0.58	11.97	51.38	2.38	277.80	0.54	-0.25	-0.44	-0.78	-0.60	-0.30	-0.70	-0.49
Others	Z8	8.52	17.01	0.80	1.24	11.15	54.18	55.63	749.70	0.80	-0.68	-0.74	0.12	-0.81	-0.26	0.15	1.63
Others	Z9	7.98	25.98	1.39	0.77	14.38	75.18	32.88	328.80	0.21	-0.29	-0.13	-0.52	0.02	0.00	-0.21	-0.26
Others	Z10	7.77	47.78	2.42	1.07	14.44	87.78	15.98	351.80	-0.01	0.67	0.93	-0.11	0.04	0.16	-0.48	-0.16
Others	Z11	8.41	20.56	0.81	0.66	11.29	30.38	2.18	260.80	0.68	-0.52	-0.73	-0.67	-0.78	-0.56	-0.70	-0.56
Others	Z14	7.80	111.70	4.51	0.84	11.82	180.18	31.13	652.20	0.02	3.48	3.08	-0.43	-0.64	1.33	-0.24	1.19
Others	Z22	8.18	31.85	1.50	0.71	9.69	59.78	11.68	310.60	0.43	-0.03	-0.01	-0.60	-1.19	-0.19	-0.55	-0.34
Others	Z38	8.51	14.85	0.70	0.74	14.74	33.18	5.83	197.70	0.79	-0.77	-0.84	-0.55	0.11	-0.53	-0.64	-0.85
ZB1	Z6	8.24	35.54	1.67	0.66	13.99	58.38	1.83	288.70	0.50	0.13	0.15	-0.67	-0.08	-0.21	-0.71	-0.44
ZB1	Z12	8.10	18.17	0.89	0.80	10.47	40.18	28.78	236.50	0.35	-0.63	-0.65	-0.48	-0.99	-0.44	-0.28	-0.67
ZB1	Z13	8.57	35.12	1.58	1.70	19.87	68.18	5.43	230.50	0.86	0.11	0.07	0.75	1.43	-0.09	-0.65	-0.70
ZB1	Z15	8.36	13.42	0.66	0.79	12.69	9.38	2.13	632.70	0.63	-0.84	-0.88	-0.49	-0.42	-0.83	-0.70	1.11
ZB1	Z16	8.28	15.69	0.80	0.89	9.82	38.78	8.33	374.90	0.54	-0.74	-0.74	-0.36	-1.16	-0.46	-0.60	-0.05
ZB1	Z17	8.46	15.80	0.73	0.58	12.10	27.58	2.93	323.90	0.74	-0.73	-0.81	-0.78	-0.57	-0.60	-0.69	-0.28
ZB1	Z18	8.09	20.49	0.63	0.47	12.90	24.78	8.83	349.40	0.33	-0.53	-0.91	-0.93	-0.36	-0.63	-0.60	-0.17
ZB1	Z19	8.42	10.82	0.46	0.77	11.79	14.98	4.83	195.30	0.69	-0.95	-1.09	-0.52	-0.65	-0.76	-0.66	-0.86
ZB1	Z20	8.40	11.32	0.64	0.67	11.08	31.78	7.93	269.30	0.67	-0.93	-0.90	-0.66	-0.83	-0.54	-0.61	-0.53

ZB1	Z21	8.67	11.42	0.50	0.51	11.50	19.18	1.48	201.40	0.97	-0.92	-1.05	-0.87	-0.72	-0.70	-0.71	-0.83
ZB1	Z23	8.25	43.41	1.25	0.85	10.63	41.58	17.93	280.20	0.51	0.48	-0.27	-0.41	-0.95	-0.42	-0.45	-0.48
ZB1	Z24	8.52	18.99	0.88	1.06	11.05	37.38	57.53	567.30	0.80	-0.59	-0.66	-0.12	-0.84	-0.47	0.18	0.81
ZB1	Z25	8.39	31.20	1.06	0.82	10.84	39.48	37.73	423.75	0.66	-0.06	-0.47	-0.45	-0.89	-0.45	-0.14	0.17
ZB1	Z26	7.87	19.89	1.12	0.76	10.78	75.18	97.63	573.30	0.10	-0.55	-0.41	-0.54	-0.91	0.00	0.82	0.84
ZB1	Z27	8.50	20.08	0.96	0.80	11.13	42.98	7.48	123.70	0.78	-0.55	-0.57	-0.48	-0.82	-0.40	-0.62	-1.18
ZB1	Z28	8.43	18.66	0.89	0.79	10.71	40.18	8.88	167.40	0.71	-0.61	-0.65	-0.50	-0.93	-0.44	-0.59	-0.98
ZB1	Z29	8.31	18.58	0.92	0.82	11.55	45.78	12.83	189.20	0.57	-0.61	-0.61	-0.45	-0.71	-0.37	-0.53	-0.89
ZB1	Z32	8.20	16.35	0.86	0.86	14.63	33.18	13.08	327.50	0.45	-0.71	-0.67	-0.40	0.08	-0.53	-0.53	-0.27
ZB1	Z35	8.27	20.54	1.03	0.86	11.80	35.98	11.05	220.75	0.53	-0.53	-0.50	-0.40	-0.64	-0.49	-0.56	-0.74
ZB1	Z36	8.63	14.53	0.79	0.87	12.16	23.38	16.58	310.60	0.92	-0.79	-0.75	-0.38	-0.55	-0.65	-0.47	-0.34
ZB1	Z42	8.28	19.52	1.23	1.96	15.92	51.38	67.13	698.70	0.54	-0.57	-0.30	1.10	0.42	-0.30	0.33	1.40
ZB1	Z44	8.51	10.37	0.56	0.84	15.70	30.38	46.68	175.90	0.79	-0.97	-0.99	-0.42	0.36	-0.56	0.01	-0.95
ZB1	Z48	5.16	60.46	2.80	3.00	7.88	128.38	305.88	684.30	-2.86	1.23	1.31	2.51	-1.66	0.67	4.14	1.34
ZB1	Z49	6.29	61.02	2.89	3.27	18.69	145.18	295.88	558.60	-1.63	1.25	1.41	2.88	1.13	0.88	3.98	0.77
ZB1	Z50	6.03	51.77	2.25	1.77	8.61	89.18	77.08	546.00	-1.91	0.85	0.76	0.84	-1.47	0.18	0.49	0.72
ZB1	Z51	5.63	109.88	4.30	0.90	13.50	208.18	21.93	327.50	-2.34	3.39	2.86	-0.34	-0.21	1.68	-0.39	-0.27
ZB1	Z54	5.83	57.75	2.65	2.84	11.73	120.91	226.28	596.30	-2.13	1.11	1.16	2.30	-0.66	0.58	2.87	0.94
ZB1	Z55	8.30	19.19	1.04	1.85	16.02	46.13	60.31	332.08	0.57	-0.58	-0.49	0.95	0.44	-0.36	0.23	-0.24
ZB2	Z30	8.27	21.03	1.22	0.96	11.59	49.98	12.18	587.70	0.53	-0.50	-0.31	-0.26	-0.70	-0.31	-0.54	0.90
ZB2	Z31	8.14	22.57	1.21	1.24	13.80	48.58	64.98	281.40	0.39	-0.44	-0.32	0.12	-0.13	-0.33	0.30	-0.47
ZB2	Z33	8.13	23.13	1.25	0.79	11.73	19.18	15.08	200.10	0.38	-0.41	-0.28	-0.49	-0.66	-0.70	-0.50	-0.84
ZB2	Z34	8.40	17.94	0.81	0.95	11.88	52.78	7.03	241.40	0.67	-0.64	-0.72	-0.27	-0.63	-0.28	-0.62	-0.65
ZB2	Z37	8.75	9.17	0.38	0.73	13.98	12.18	5.08	211.10	1.05	-1.02	-1.17	-0.57	-0.08	-0.79	-0.66	-0.79
ZB2	Z39	8.35	10.40	0.49	0.83	13.34	608.58	10.08	256.00	0.62	-0.97	-1.06	-0.43	-0.25	6.72	-0.58	-0.59
ZB2	Z40	8.26	64.61	3.27	1.05	14.26	115.78	10.73	618.60	0.52	1.41	1.80	-0.14	-0.01	0.51	-0.57	1.04

ZB2	Z41	8.44	12.33	0.60	1.00	15.39	24.78	46.38	807.90	0.72	-0.89	-0.94	-0.20	0.28	-0.63	0.00	1.89
ZB2	Z43	8.21	19.58	0.94	1.96	16.61	35.98	83.63	349.40	0.47	-0.57	-0.59	1.10	0.59	-0.49	0.60	-0.17
ZB2	Z45	8.21	27.31	1.43	1.01	15.85	66.78	43.83	104.30	0.47	-0.23	-0.08	-0.20	0.40	-0.10	-0.04	-1.27
ZB2	Z46	8.02	5.83	0.44	0.71	15.04	13.58	0.58	41.20	0.26	-1.17	-1.11	-0.60	0.19	-0.77	-0.73	-1.55
ZB2	Z47	7.31	37.17	2.01	1.28	14.86	98.98	99.38	343.30	-0.51	0.20	0.51	0.18	0.14	0.30	0.85	-0.19
ZB2	Z53	7.26	31.57	1.41	0.90	26.11	117.18	61.03	570.90	-0.57	-0.04	-0.11	-0.35	3.04	0.53	0.24	0.83
ZA	Z52	7.16	32.05	1.62	0.43	17.23	77.98	1.23	299.60	-0.68	-0.02	0.11	-0.98	0.76	0.04	-0.72	-0.39
ZA	Z56	7.22	104.63	4.95	5.04	15.62	262.78	190.38	982.50	-0.61	3.16	3.53	5.29	0.34	2.37	2.30	2.68
ZA	Z57	5.63	21.06	1.42	1.15	10.72	192.78	94.88	1095.60	-2.34	-0.50	-0.10	0.00	-0.93	1.48	0.78	3.19
ZA	Z58	6.80	22.40	1.09	0.52	16.16	77.98	3.18	101.90	-1.07	-0.44	-0.44	-0.86	0.48	0.04	-0.69	-1.28
ZA	Z59	8.09	24.31	1.32	1.29	17.10	52.78	32.88	642.30	0.33	-0.36	-0.21	0.18	0.72	-0.28	-0.21	1.15
ZA	Z60	7.46	18.25	1.11	1.25	17.23	48.58	13.48	243.80	-0.35	-0.63	-0.41	0.14	0.75	-0.33	-0.52	-0.64
ZA	Z61	4.02	52.93	2.35	0.93	15.88	104.58	134.13	334.80	-4.10	0.90	0.86	-0.30	0.41	0.37	1.40	-0.23
ZA	Z62	7.93	24.47	1.31	2.71	18.13	56.98	61.48	283.90	0.16	-0.35	-0.21	2.12	0.99	-0.23	0.24	-0.46
ZA	Z63	7.79	12.76	0.48	0.79	21.05	44.38	20.13	155.30	0.01	-0.87	-1.07	-0.50	1.74	-0.39	-0.42	-1.04
ZA	Z64	7.86	18.62	0.89	1.22	19.59	50.68	40.80	219.60	0.08	-0.61	-0.64	0.09	1.36	-0.31	-0.09	-0.75
ZA	Z65	7.95	62.90	3.05	1.56	24.08	91.98	31.38	533.10	0.18	1.33	1.57	0.55	2.52	0.21	-0.24	0.66
ZA	Z66	7.91	27.79	1.40	1.06	25.51	44.38	19.58	239.00	0.14	-0.21	-0.12	-0.12	2.89	-0.39	-0.42	-0.66
ZA	Z67	7.93	45.34	2.22	1.21	24.80	68.18	25.48	386.05	0.16	0.56	0.72	0.08	2.70	-0.09	-0.33	0.00
ZA	Z68	7.77	72.82	3.20	1.44	13.98	140.98	90.38	746.10	-0.01	1.77	1.73	0.40	-0.08	0.83	0.70	1.62
ZA	Z69	7.41	64.64	2.48	0.99	10.46	59.78	4.28	92.20	-0.41	1.41	0.99	-0.22	-0.99	-0.19	-0.67	-1.32
ZA	Z70	7.75	32.10	2.14	1.24	12.76	69.58	83.63	630.30	-0.04	-0.02	0.64	0.11	-0.40	-0.07	0.60	1.09
ZA	Z71	6.85	42.31	2.06	0.81	21.23	105.98	4.33	550.50	-1.02	0.43	0.56	-0.46	1.79	0.39	-0.67	0.74
ZA	Z72	6.06	40.94	1.69	1.13	11.88	68.18	84.38	209.90	-1.88	0.37	0.18	-0.03	-0.62	-0.09	0.61	-0.79

ZA, samples with green pericarps (n=18) derived from group of *Z, armatum*; ZB1, samples with red pericarps from Hancheng (n=28) derived from group of *Z. bungeanum*; ZB2, samples with red pericarps from Fengxian (n=13) derived from group of *Z. bungeanum*; Others, samples with red pericarps, a class of several species (n=13), excluding *Z. bungeanum*.

Table S3 Raw data and transformed (Z-Score) values of potentially toxic elements in prickly ash soils.

	Code	Raw data						Transformed (Z-Score) values					
		Al (g kg^{-1})	As (mg kg^{-1})	Cd (mg kg^{-1})	Pb (mg kg^{-1})	Mn (mg kg^{-1})	Ni (mg kg^{-1})	Al	As	Cd	Pb	Mn	Ni
Others	ZB1	52.35	10.63	0.42	13.13	544.83	18.88	-0.17	1.06	-0.03	0.01	0.25	-0.2
Others	ZB2	79.58	4.83	0.18	19	502.92	18.83	1.18	-1.16	-0.78	0.44	0.06	-0.21
Others	ZB3	63.71	9.02	0.43	25.94	808.41	38.6	0.39	0.44	0.01	0.94	1.42	1.73
Others	ZB4	69.2	5.79	0.3	14.99	391.92	13.71	0.67	-0.8	-0.42	0.14	-0.43	-0.71
Others	ZB5	33.7	7.5	0.29	9.79	487.87	16.66	-1.1	-0.14	-0.46	-0.24	-0.01	-0.42
Others	ZB7	30.99	9.17	0.32	7.78	429.67	20.39	-1.24	0.5	-0.35	-0.38	-0.27	-0.06
Others	ZB8	29.41	9.25	0.37	7.86	443.99	18.79	-1.31	0.53	-0.2	-0.38	-0.2	-0.21
Others	ZB9	23.83	8.9	0.35	8.41	509.77	21.24	-1.59	0.39	-0.27	-0.34	0.09	0.03
Others	ZB10	34.69	9.82	0.43	8.18	510.31	17.01	-1.05	0.75	-0.02	-0.35	0.09	-0.39
Others	ZB11	46.13	8.32	0.31	6.76	397.02	16.03	-0.48	0.17	-0.38	-0.46	-0.41	-0.48
Others	ZB14	37.86	10.11	1.26	42.68	394.36	18.21	-0.89	0.86	2.61	2.16	-0.42	-0.27
Others	ZB22	25.96	8.65	0.32	5.65	388.86	16.38	-1.49	0.3	-0.35	-0.54	-0.45	-0.45
Others	ZB38	43.64	9.32	0.37	6.3	411.28	16.75	-0.61	0.56	-0.19	-0.49	-0.35	-0.41
ZB1	Z6	35.05	7.34	0.24	6.67	566.59	19.57	-1.03	-0.2	-0.6	-0.46	0.34	-0.13
ZB1	Z12	56.64	8.77	0.3	6.26	398.7	16.59	0.04	0.34	-0.42	-0.49	-0.4	-0.43
ZB1	Z13	58.13	8.99	0.33	7.29	297.71	21.12	0.12	0.43	-0.31	-0.42	-0.85	0.02
ZB1	Z15	37.34	8.05	0.3	5.74	345.43	14.62	-0.92	0.07	-0.41	-0.53	-0.64	-0.62
ZB1	Z16	40.91	7.77	0.44	19.85	367.38	15.3	-0.74	-0.04	0.04	0.5	-0.54	-0.55
ZB1	Z17	61.27	8.05	0.33	7.12	369.02	16.44	0.27	0.07	-0.31	-0.43	-0.54	-0.44
ZB1	Z18	30.24	9.3	0.38	8.14	617.14	27.66	-1.27	0.55	-0.16	-0.36	0.57	0.66
ZB1	Z19	33.4	8.9	0.33	6.99	656.02	22.97	-1.12	0.39	-0.3	-0.44	0.74	0.2
ZB1	Z20	54.62	8.02	0.28	4.65	356.74	14.13	-0.06	0.06	-0.48	-0.61	-0.59	-0.67
ZB1	Z21	86.02	8.35	0.3	5.12	386.36	17.5	1.51	0.18	-0.4	-0.58	-0.46	-0.34

ZB1	Z23	37.14	8.33	0.34	10.29	378.29	16.62	-0.93	0.18	-0.29	-0.2	-0.5	-0.42
ZB1	Z24	52.94	8.74	0.36	7.38	436.41	17.63	-0.14	0.33	-0.22	-0.41	-0.24	-0.33
ZB1	Z25	45.04	8.53	0.35	8.84	407.35	17.12	-0.54	0.25	-0.26	-0.3	-0.37	-0.37
ZB1	Z26	61.86	9.35	0.39	8.76	452.7	19.82	0.3	0.57	-0.13	-0.31	-0.16	-0.11
ZB1	Z27	49.63	8.24	0.34	6.85	409.94	17.94	-0.31	0.14	-0.3	-0.45	-0.35	-0.29
ZB1	Z28	47.05	8.41	0.35	6.28	412.76	17.34	-0.44	0.21	-0.27	-0.49	-0.34	-0.35
ZB1	Z29	56.54	8.85	0.37	6.67	426.04	18.26	0.04	0.37	-0.19	-0.46	-0.28	-0.26
ZB1	Z32	57.43	8.91	0.4	7.21	452.8	18.98	0.08	0.4	-0.1	-0.42	-0.16	-0.19
ZB1	Z35	63.82	8.82	0.31	8	385	19	0.4	0.37	-0.38	-0.37	-0.47	-0.19
ZB1	Z36	59.22	9.54	0.31	6.79	371.71	22.21	0.17	0.64	-0.38	-0.45	-0.52	0.12
ZB1	Z42	66.68	8.2	0.35	7.17	417.78	17.64	0.54	0.13	-0.27	-0.43	-0.32	-0.32
ZB1	Z44	62.71	9.65	0.92	14.15	413.44	17.15	0.34	0.68	1.55	0.08	-0.34	-0.37
ZB1	Z48	87.85	4.24	0.08	2.03	607.14	63.44	1.6	-1.39	-1.09	-0.8	0.52	4.16
ZB1	Z49	40.36	2.15	0.15	42.96	267.7	10.62	-0.77	-2.19	-0.89	2.18	-0.99	-1.01
ZB1	Z50	47.1	9.18	0.23	3.11	401.5	74.65	-0.43	0.5	-0.63	-0.72	-0.39	5.26
ZB1	Z51	21.12	10.13	0.77	4.12	606.64	17.92	-1.73	0.86	1.07	-0.65	0.52	-0.3
ZB1	Z54	79.35	11.85	1.36	26.85	510.18	41.75	1.17	1.53	2.93	1.01	0.09	2.04
ZB1	Z55	95.39	2.7	0.15	0.98	526.53	21.99	1.97	-1.98	-0.88	-0.88	0.16	0.1
ZB2	ZB30	41.34	8.7	0.4	7.96	484	19.79	-0.72	0.32	-0.11	-0.37	-0.02	-0.11
ZB2	ZB31	65.85	9.61	0.43	7.27	476.73	20.7	0.5	0.67	-0.01	-0.42	-0.06	-0.02
ZB2	ZB33	47.1	8.55	0.27	5.41	371.42	15.04	-0.43	0.26	-0.5	-0.55	-0.53	-0.58
ZB2	ZB34	47.84	8.22	0.02	14.54	466.03	18.74	-0.4	0.14	-1.31	0.11	-0.1	-0.22
ZB2	ZB37	24.24	8.86	0.31	4.78	384.93	15.01	-1.57	0.38	-0.38	-0.6	-0.47	-0.58
ZB2	ZB39	29.4	8.69	0.34	5.84	383.45	15.37	-1.32	0.31	-0.3	-0.52	-0.47	-0.55
ZB2	ZB40	53.59	8.56	0.4	8.05	430.59	17.76	-0.11	0.26	-0.1	-0.36	-0.26	-0.31
ZB2	ZB41	65.58	9	0.33	5.58	403.77	16.61	0.49	0.43	-0.32	-0.54	-0.38	-0.43
ZB2	ZB43	70.33	10.04	0.4	9.15	423.29	17.09	0.72	0.83	-0.08	-0.28	-0.29	-0.38

ZB2	ZB45	66.57	9.49	0.36	5.38	446.02	17.77	0.54	0.62	-0.22	-0.56	-0.19	-0.31
ZB2	ZB46	55.67	14.1	0.82	6.85	372.93	24.05	-0.01	2.39	1.23	-0.45	-0.52	0.3
ZB2	ZB47	66.64	8.04	0.26	5.09	361.71	17.57	0.54	0.07	-0.54	-0.58	-0.57	-0.33
ZB2	ZB53	74.67	8.32	0.59	67.15	842.09	16.58	0.94	0.17	0.5	3.95	1.57	-0.43
ZA	Z52	33.11	5.67	0.44	27.76	306.2	20.59	-1.13	-0.84	0.04	1.07	-0.82	-0.03
ZA	Z56	61.7	9.16	0.72	52.16	486.44	13.56	0.29	0.49	0.92	2.85	-0.01	-0.72
ZA	Z57	71.42	1.69	0.29	6.91	398.1	18.58	0.78	-2.36	-0.43	-0.44	-0.41	-0.23
ZA	Z58	90.18	3.24	0.25	7.35	402.97	24.65	1.71	-1.77	-0.59	-0.41	-0.39	0.36
ZA	Z59	47.27	4.84	0.32	8.57	397.15	18.8	-0.42	-1.16	-0.35	-0.32	-0.41	-0.21
ZA	Z60	42.29	5.59	0.4	8.08	569.06	21.76	-0.67	-0.87	-0.09	-0.36	0.35	0.08
ZA	Z61	27.22	3.78	0.26	13.66	311.15	14.44	-1.42	-1.57	-0.54	0.05	-0.79	-0.64
ZA	Z62	72.03	4.41	0.44	6.92	536.36	19.34	0.81	-1.32	0.04	-0.44	0.21	-0.16
ZA	Z63	80.93	2.25	0.26	5.51	478.94	22.22	1.25	-2.15	-0.55	-0.55	-0.05	0.12
ZA	Z64	80.93	2.25	0.26	5.51	478.94	22.22	1.25	-2.15	-0.55	-0.55	-0.05	0.12
ZA	Z65	59.99	8.61	0.62	27.09	513.79	12.05	0.21	0.28	0.6	1.03	0.11	-0.87
ZA	Z66	96.1	5.82	0.31	12.75	686.83	26.19	2.01	-0.78	-0.39	-0.02	0.88	0.51
ZA	Z67	74.67	8.32	0.59	67.15	842.09	16.58	0.94	0.17	0.5	3.95	1.57	-0.43
ZA	Z68	27.59	2.59	0.55	41.11	872.44	13.06	-1.41	-2.02	0.39	2.05	1.71	-0.77
ZA	Z69	95.39	2.7	0.15	0.98	526.53	21.99	1.97	-1.98	-0.88	-0.88	0.16	0.1
ZA	Z70	82.32	12.76	2.1	18.68	2059.73	40.7	1.32	1.87	5.25	0.41	6.99	1.94
ZA	Z71	79.35	11.85	1.36	26.85	510.18	41.75	1.17	1.53	2.93	1.01	0.09	2.04
ZA	Z72	90.52	10.2	0.67	14.47	553.52	20.31	1.73	0.89	0.77	0.11	0.29	-0.06

ZA, samples with green pericarps (n=18) derived from group of *Z, armatum*; ZB1, samples with red pericarps from Hancheng (n=28) derived from group of *Z. bungeanum*; ZB2, samples with red pericarps from Fengxian (n=13) derived from group of *Z. bungeanum*; Others, samples with red pericarps, a class of several species (n=13), excluding *Z. bungeanum.*

Table S4 Transformed (Z-Score) values of alkylamides data in different prickly ash pericarps.

Group	Code	Tetrahydrobungeanool	ZP- amide E	ZP- amide A	ZP- amide B	(2E,7E,9E)-N-(2-hydroxy-2-methylpropyl)-6,11-dioxo-2,7,9-dodecatrienamide	ZP-amide C	ZP-amide D	Hydroxyl-α-sanschool	Hydroxyl-β-sanschool
Others	Z1	0.10	1.96	-1.26	-1.54	1.53	0.10	-1.19	0.38	1.38
Others	Z2	-0.98	-0.91	-0.49	-0.82	1.07	-0.85	-0.12	0.27	1.36
Others	Z3	0.37	2.17	-1.22	-1.40	1.55	1.44	-1.19	0.26	0.38
Others	Z4	-1.51	-0.91	-1.78	-2.27	-1.23	-2.15	-1.19	-6.77	-0.12
Others	Z5	-0.15	1.31	0.55	0.45	-0.43	-0.11	-1.19	0.23	1.18
Others	Z7	-0.26	1.53	0.74	0.87	-0.35	0.10	-1.19	-0.33	-1.44
Others	Z8	0.90	1.78	1.03	1.11	0.05	4.66	-1.19	0.15	1.66
Others	Z9	-0.52	0.58	0.99	0.84	-0.48	-0.95	-1.19	-2.35	-0.87
Others	Z10	-0.58	0.42	1.75	1.52	0.07	-0.24	-1.19	-0.78	1.35
Others	Z11	-0.21	-0.91	0.15	0.34	1.74	-0.01	-1.19	-1.55	-0.54
Others	Z14	-0.08	-0.91	2.97	3.43	0.12	1.13	-1.19	0.20	0.15
Others	Z22	-1.10	-0.91	0.40	0.42	-0.72	-0.07	-1.19	-0.61	-0.01
Others	Z38	-0.58	-0.72	-0.41	-0.54	-0.76	-0.51	0.18	0.28	0.15
ZB1	Z6	0.28	0.38	0.12	0.10	-0.43	0.68	-1.19	0.25	0.47
ZB1	Z12	-0.08	0.70	-0.44	-0.63	2.17	-0.17	-1.19	-0.07	-0.40
ZB1	Z13	0.52	0.70	-1.51	-0.33	-0.79	0.01	-1.19	0.50	0.33
ZB1	Z15	0.07	0.45	0.34	0.28	-0.32	0.63	-1.19	0.20	0.17
ZB1	Z16	0.17	0.40	-1.49	0.19	-1.01	-0.18	-1.19	0.29	-1.01
ZB1	Z17	-0.61	0.63	0.27	0.07	-0.71	-0.49	-1.19	0.24	-1.29
ZB1	Z18	-0.46	-0.91	-0.17	-0.25	1.29	-0.20	-1.19	0.37	-0.09
ZB1	Z19	-0.72	-0.71	-0.79	-1.01	0.04	0.08	-1.19	0.46	-0.05
ZB1	Z20	0.11	3.44	-1.46	-1.73	-0.81	-1.09	-1.19	0.20	-0.61
ZB1	Z21	-0.20	-0.91	0.41	0.26	-0.50	1.30	-1.19	0.14	0.90

ZB1	Z23	0.24	-0.75	-0.27	-0.17	3.17	1.02	0.44	0.16	-1.10
ZB1	Z24	-1.14	0.80	0.44	0.23	-0.41	-0.82	1.02	-0.39	-1.39
ZB1	Z25	-0.36	-0.81	0.22	0.15	1.08	0.70	0.26	0.41	-0.75
ZB1	Z26	0.06	0.18	0.00	-0.11	-0.58	0.03	0.88	0.16	-0.41
ZB1	Z27	-0.33	-0.91	0.40	0.54	-0.40	0.62	0.51	0.32	-1.07
ZB1	Z28	-0.57	-0.81	-0.49	-0.67	2.77	-0.41	0.42	0.45	-1.00
ZB1	Z29	2.67	0.47	-1.52	-0.12	-1.13	-0.24	0.37	0.51	-1.22
ZB1	Z32	0.61	0.49	0.33	0.27	-0.31	0.59	1.12	0.18	0.06
ZB1	Z35	-0.34	-0.91	0.13	0.06	-0.34	-2.33	0.79	0.13	-1.44
ZB1	Z36	-0.55	-0.56	-0.24	-0.30	-0.63	-0.23	0.43	0.20	0.25
ZB1	Z42	-0.31	-0.91	-1.09	-1.39	-0.95	-0.79	0.17	0.30	0.78
ZB1	Z44	-0.55	-0.91	0.46	0.28	2.17	0.03	0.55	0.05	0.75
ZB1	Z48	-0.38	-0.91	-0.90	-0.94	-0.48	-0.68	-0.19	0.53	-0.66
ZB1	Z49	-0.04	-0.75	-0.38	-0.34	-0.19	0.76	0.29	0.64	-0.45
ZB1	Z50	2.12	-0.91	0.06	-0.29	-0.79	-0.83	0.15	0.12	0.58
ZB1	Z51	-0.13	-0.91	-1.10	-1.36	-0.99	-2.33	-0.13	0.43	-0.67
ZB1	Z54	-0.41	-0.49	1.15	0.92	-0.23	0.97	0.80	0.42	0.14
ZB1	Z55	-0.68	0.05	0.76	1.19	-0.62	0.09	0.76	0.02	0.01
ZB2	Z30	1.93	-0.20	0.43	0.11	-0.32	0.77	0.80	0.48	-0.42
ZB2	Z31	0.18	-0.91	-0.56	-0.64	1.86	0.42	0.01	0.18	-1.02
ZB2	Z33	2.54	0.39	1.61	1.09	-0.07	0.90	0.92	-0.04	0.09
ZB2	Z34	-0.14	1.05	-0.14	-0.36	1.46	0.32	0.57	0.17	2.76
ZB2	Z37	-0.45	-0.63	0.00	-0.52	0.23	-0.48	-0.27	0.53	-0.36
ZB2	Z39	1.99	0.28	-1.59	-2.09	-0.14	0.46	-0.25	0.33	-1.06
ZB2	Z40	-0.55	-0.42	-0.30	-0.53	0.29	-0.35	0.27	0.31	0.73
ZB2	Z41	1.55	0.00	0.43	0.88	-0.49	1.36	-0.32	0.25	0.41

ZB2	Z43	-0.30	-0.39	0.42	0.08	-0.50	0.07	0.63	0.16	0.40
ZB2	Z45	2.23	0.43	-1.60	-0.64	-1.10	1.20	-0.06	1.00	-1.13
ZB2	Z46	-0.01	-0.91	-0.31	-0.68	2.16	0.49	-1.19	0.09	-0.68
ZB2	Z47	-0.97	-0.91	0.58	0.58	-0.14	0.04	0.49	0.45	-0.74
ZB2	Z53	0.11	-0.21	0.17	0.20	-0.43	0.11	1.10	0.18	0.97
ZA	Z52	-0.50	-0.51	-0.90	-1.18	-0.06	-0.75	-0.30	0.16	-0.20
ZA	Z56	1.63	0.73	-0.80	-1.06	-0.85	-0.48	-0.23	0.11	0.02
ZA	Z57	-0.83	-0.91	0.30	0.39	-0.18	-0.91	0.69	-0.09	0.41
ZA	Z58	-0.12	1.04	1.70	2.28	0.59	1.39	2.71	0.11	2.41
ZA	Z59	-1.08	-0.35	1.80	1.70	0.82	0.55	2.50	-0.65	0.93
ZA	Z60	-0.42	-0.91	0.21	0.31	-0.42	-2.33	0.11	0.49	-1.06
ZA	Z61	3.48	-0.91	-1.58	0.40	-1.20	0.78	0.04	0.45	-0.47
ZA	Z62	0.35	-0.91	-1.41	-0.76	-1.11	0.12	-1.02	-2.77	-1.22
ZA	Z63	-0.86	-0.91	1.71	1.59	0.50	0.08	2.25	-0.41	0.65
ZA	Z64	-1.15	0.11	0.72	0.52	-0.17	-0.10	1.24	0.48	2.75
ZA	Z65	-1.14	0.80	0.44	0.23	-0.41	-0.82	1.02	-0.39	-1.37
ZA	Z66	-0.62	1.03	-0.88	-1.16	-1.01	-0.90	-0.37	0.23	0.60
ZA	Z67	1.30	2.09	-0.33	-0.60	-0.42	0.06	0.08	0.97	2.58
ZA	Z68	-0.21	2.33	-0.31	-0.52	-0.68	-0.14	0.07	0.49	-0.23
ZA	Z69	-0.29	0.84	2.56	2.19	0.51	-0.07	1.85	0.12	-0.50
ZA	Z70	-0.51	0.16	0.55	0.56	-0.26	0.04	1.10	0.28	-0.49
ZA	Z71	-1.08	-0.46	0.24	0.23	-0.43	-0.68	0.88	-0.07	-0.19
ZA	Z72	-0.44	-0.91	0.18	0.06	-0.28	-0.43	0.93	-0.22	0.01

ZA, samples with green pericarps (n=18) derived from group of *Z, armatum*; ZB1, samples with red pericarps from Hancheng (n=28) derived from group of *Z. bungeanum*; ZB2, samples with red pericarps from Fengxian (n=13) derived from group of *Z. bungeanum*; Others, samples with red pericarps, a class of several species (n=13), excluding *Z. bungeanum*; ZP in alkylamides represents *Z. piperitum*.
